# Exosomal MicroRNAs modulate the cognitive function in fasudil treated APPswe/PSEN1dE9 transgenic (APP/PS1) mice model of Alzheimer’s disease

**DOI:** 10.1007/s11011-024-01395-8

**Published:** 2024-08-01

**Authors:** Yuqing Yan, Ye Gao, Gajendra Kumar, Qingli Fang, Hailong Yan, Nianping Zhang, Yuna Zhang, Lijuan Song, Jiehui Li, Yucheng Zheng, Nan Zhang, Peijun Zhang, Cungen Ma

**Affiliations:** 1 Institute of Brain Science, Shanxi Key Laboratory of Inflammatory Neurodegenerative Diseases, Medical School of Shanxi Datong University, Datong, China; 2 The Key Research Laboratory of Benefiting Qi for Acting Blood Circulation Method to Treat Multiple, Sclerosis of State Administration of Traditional Chinese Medicine, Research Center of Neurobiology, Shanxi University of Chinese Medicine, Taiyuan, China; 3 Department of Neuroscience, City University of Hong Kong, Kowloon, Hong Kong

**Keywords:** Alzheimer’s disease, Exosome, Fasudil, miRNA, APPswe/PSEN1dE9 transgenic (APP/PgeS1) mice

## Abstract

Alzheimer’s disease (AD) is characterized by cognitive decline stemming from the accumulation of beta-amyloid (Aβ) plaques and the propagation of tau pathology through synapses. Exosomes, crucial mediators in neuronal development, maintenance, and intercellular communication, have gained attention in AD research. Yet, the molecular mechanisms involving exosomal miRNAs in AD remain elusive. In this study, we treated APPswe/PSEN1dE9 transgenic (APP/PS1) mice, a model for AD, with either vehicle (ADNS) or fasudil (ADF), while C57BL/6 (control) mice received vehicle (WT). Cognitive function was evaluated using the Y-maze test, and AD pathology was confirmed through immunostaining and western blot analysis of Aβ plaques and phosphorylated tau. Exosomal RNAs were extracted, sequenced, and analyzed from each mouse group. Our findings revealed that fasudil treatment improved cognitive function in AD mice, as evidenced by increased spontaneous alternation in the Y-maze test and reduced Aβ plaque load and phosphorylated tau protein expression in the hippocampus. Analysis of exosomal miRNAs identified three miRNAs (mmu-let-7i-5p, mmu-miR-19a-3p, mmu-miR-451a) common to both ADNS vs ADF and WT vs ADNS groups. Utilizing miRTarBase software, we predicted and analyzed target genes associated with these miRNAs. Gene ontology (GO) annotation and Kyoto Encyclopedia of Genes and Genomes (KEGG) pathway analysis of miRNA target genes indicated that mmu-miR-19a-3p and mmu-miR-451a are implicated in signal transduction, immune response, cellular communication, and nervous system pathways. Specifically, mmu-miR-19a-3p targeted genes involved in the sphingolipid signaling pathway, such as Pten and Tnf, while mmu-miR-451a targeted Nsmaf, Gnai3, and Akt3. Moreover, mmu-miR-451a targeted Myc in signaling pathways regulating the pluripotency of stem cells. In conclusion, fasudil treatment enhanced cognitive function by modulating exosomal MicroRNAs, particularly mmu-miR-451a and mmu-miR-19a-3p. These miRNAs hold promise as potential biomarkers and therapeutic targets for novel AD treatments.

**Graphical Abstract:**

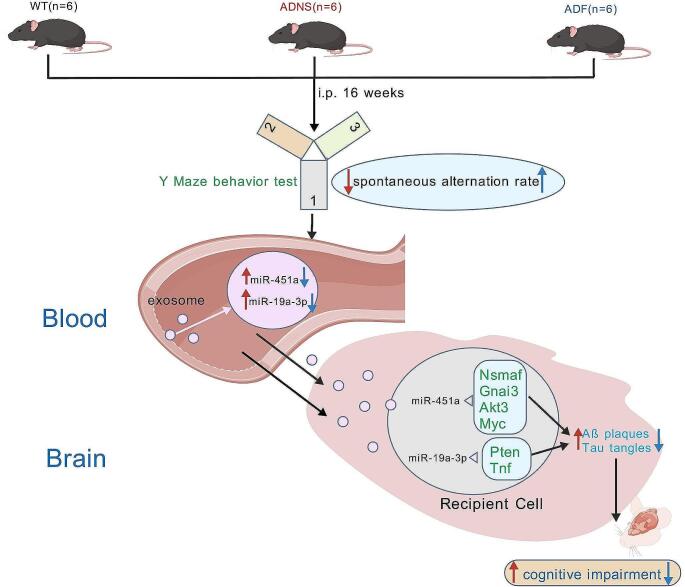

**Supplementary Information:**

The online version contains supplementary material available at 10.1007/s11011-024-01395-8.

## Introduction

Alzheimer's disease (AD) is a progressive neurodegenerative condition characterized by cognitive decline and the accumulation of beta-amyloid (Aβ) plaques and hyperphosphorylated tau tangles. These hallmark pathological biomarkers, Aβ and p-tau, are central to AD pathology (Van Giau and An 2016). Extracellular vesicles (EVs) have emerged as promising tools for diagnosis and therapeutic guidance in AD. These vesicles, capable of traversing the blood–brain barrier, offer a potential source of AD biomarkers. Numerous studies have indicated that EVs harbor misfolded proteins associated with AD pathology (Liu et al. 2019; Nielsen et al. 2021; Sharma 2022). EVs include exosomes, microvesicles and apoptotic bodies that are known to participate in intercellular communication (Paschon et al. 2016). Exosomes have been given attention due to their role as pathological and therapeutic cargo. Exosome vesicles are approximately 30–100 nm in diameter with a lipid bilayer membrane structure, released upon the exocytosis of a multivesicular body. Distant intercellular communication occurs through RNA signals triggered by pathogenic materials of AD during development and progression (Zhou et al. 2018; Guo et al. 2020). Pathogenic materials such as proteins, micro-RNAs, and messenger RNAs, are invaginated in exosomes and released (Bunggulawa et al. 2018). MicroRNAs (miRNAs) are a family of small non-coding RNA molecules reported to be involved in pathogenesis of neurodegenerative diseases and CNS injuries (Gayen et al. 2020). Exosomes carry these MicroRNAs (miRNAs) for cell–cell communication in neurons (Wang et al. 2020). Exosomes transport miRNA, mRNA, and transcription factors (TFs) to neighboring cells and influence their cellular function (Yan et al. 2019). Exosomes represent several common proteins of cytotypes such as CD63, CD81, CD9, TSG-101, ALIX, and HSP70 and these markers aid in the identification of cell type (Manna et al. 2020). Exosome-derived MicroRNAs (miRNAs) and long non-coding RNAs (lncRNAs) are involved in the pathophysiology of cancers and neurodegenerative diseases (Beeraka et al. 2020). Exosomal miRNAs (ex-miRNAs) are highly stable, resistant to degradation and they served as reliable biomarkers for the early clinical diagnosis of AD (Dong et al. 2020).

Exosomes in blood are a novel source of biomarkers for the diagnosis and prognosis of diseases (Helwa et al. 2017). Aβ deposition and inflammation pathway have been implicated in AD pathology and progression. Inflammatory cytokines are released from exosomes after being triggered by sensitive cells (Jung et al. 2020). A recent study showed an increased level of cytokines in the mice, which was immunized with pooled exosomes isolated from the sera of lung transplant recipients (LTxR) with bronchiolitis obliterans syndrome (BOS) (Gunasekaran et al. 2018). Inflammatory markers are non-specific due to pooling of samples during analysis, however, analysis of exosomal miRNA offers high specificity and sensitivity to predict the biomarkers of AD (Zhang et al. 2018). Exosomes carrying proteins, RNA, and DNA are derived from their originating cells and protected from degradation in the circulation (Capello et al. 2019). Mesenchymal stem cells (MSCs) -derived exosomes contain high levels of miR-133b and miRNAs in the miR-17 ~ 92 cluster and repress the expression of PTEN, CTGF and RhoA to remodel the neurites and improve the functional recovery in a mouse model of stroke (Ma et al. 2019). Exosomes isolated from pooled serum samples of patients and healthy controls have been used to analyze the protein profiles (Ding et al. 2020). Exosomes have been shown to establish communication between cells and exchange genetic materials (Wang et al. 2013) such as mRNA, miRNA, transfer RNAs and long non-coding RNAs (lncRNAs) (Kumar et al. 2020) to modulate the physiological processes such as immune response, inflammatory response, angiogenesis, apoptosis, blood coagulation, and cell debris disposal (He et al. 2021). Cell type specificity of exosomes with RNA and protein components has been proposed as potential early diagnostic markers for several diseases and offers a targeted carrier of drugs for treatment (Xia et al. 2019).

The current investigation attempts to elucidate the unique expression patterns of miRNAs within exosomes extracted from the serum of APP/PS1 mice subjected to fasudil treatment. Our objective is to underscore the potential of exosomal miRNA-based approaches for diagnosing and treating Alzheimer's disease, offering innovative therapeutic avenues.

## Materials and methods

### Animals and treatment

Male APP /PS1 mice (8 months old) expressing human amyloid precursor protein (HuAPP695swe) and a mutant human presenilin 1 (PS1-dE9) were purchased from Beijing Huafukang Bioscience CO., LTD (HFK, Beijing, China). All animals were housed in the pathogen-free facilities at the Institute of Brain Science, Shanxi Datong University and maintained at constant room temperature (25 ± 2^0^C) and humidity (50 ± 5%) in a 12-h light/12-h dark cycle. APP/PS1 mice were pre-screened based on the normal physiological behavior and randomly divided into two treatment groups: 1) vehicle-treated mice (ADNS); received normal saline (volume was adjusted similarly to fasudil treatment); 2) fasudil-treated (ADF) mice; received a daily injection of fasudil (Tianjin Chase Sun Pharmaceutical Co., Ltd.), 25 mg/kg/day, i.p., 16 weeks). Aged and gender-matched C57BL/6 (WT) mice were used as normal controls, which received saline in the same volume (*n* = 6 per group). Animals had ad libitum access to food and water. All the experiments were performed in the compliance with the guidelines and regulations of the Administration Office of the International Council for Laboratory Animal Science. The experimental protocols were approved by the Animal Ethics Committee of Shanxi Datong University, Datong, China.

### Y Maze alternating behavior test

Y maze test measures the spatial working memory of animals. It consists of two stages and was performed at 1 h intervals. The first stage was the training period, and the mouse was given limited food and water in one arm of Y maze, while the other arm served as an experimental with reward. The new arm (left arm) was blocked by a barrier. Mouse was kept at the starting point of the arm and allowed to move freely for 10 min. After the training, the mice were put back into the home cage. The second stage was the acquisition stage, where food or water was removed from the experimental arm. The new arm barrier (left arm) was removed, and the mice were placed at the starting point and allowed to move freely for 5 min. Total time spent and the number of entries in each arm were recorded by a video tracking system (SMART V3.0 system, Panlab, Barcelona, Spain)). The apparatus was cleaned with ethanol at the end of each training or test to prevent interference due to residual (Xu et al. 2018).

### Immunofluorescence staining and western blot for Aβ plaques and phospho-Tau

Mice were sacrificed at the end of treatment. The hippocampus tissues were collected from all groups and were fixed in 4% cold paraformaldehyde for 30 min at room temperature. The sections were washed, permeabilized with 0.1% Triton X-100 for 15 min, rinsed with PBS and blocked with 1% bovine serum albumin (BSA) for 30 min at room temperature. Sections were subsequently incubated with anti-Tau (phospho S404) (dilution of 1:200, abcam, ab92676, USA) with mouse monoclonal primary antibody, anti-Aβ1-42 (dilution of 1:500, Servicebio, GB111197, China) with rabbit monoclonal primary antibody at 4ºC overnight. Slides were washed 3 times with PBS and incubated with FITC or Cy3-conjugated secondary antibodies (Invitrogen, USA) for 1 h. at RT, and then thoroughly washed with PBS for 3 times. The negative control sections were treated using the same protocols without primary antibodies. The average values of six sections from each slice were analyzed for the final analysis. Three samples were analyzed in each group. The modified coverslips were mounted onto glass slides and observed with confocal microscopy (Olympus FV1000, Japan). Images were taken at 10x (scale = 200 μm). The area (polygon) of positive cells was quantitatively analyzed by Image-Pro Plus software. All images taken for quantification were blinded during analysis.

Hippocampus proteins were transferred onto PVDF membranes and probed with ACTIN (1:1000 dilutions; Servicebio company, no. GB11001), Tau (1:1000 dilutions; Servicebio company, no. GB11178), P-Tau (1:1000 dilutions; Servicebio company, no. GB113883) and β Amyloid (1:1000 dilutions; Abcam company, no. 25524–1-AP) primary antibodies and developed by secondary antibodies, HRP reagent (Millipore, USA).

### Methods for extraction and purification of exosomes (serum)

In this study, six mice were allocated in each experimental group and blood samples were obtained by retro-orbital vein puncture. Following blood collection, serum from three mice was mixed to make two biological replicates. Subsequently, exosome isolation procedures were performed. Initially, the serum was diluted with precooled 1 × phosphate-buffered saline (PBS). Subsequently, Blood PureExo Solution (BPS) was added to the diluted serum, and the mixture was incubated at 2–8 °C for a duration of 2 h to facilitate exosome precipitation. After the incubation period, the sample underwent centrifugation at 10,000 g for 60 min at 4 °C, resulting in the separation of exosome-rich supernatant. To further enrich exosome particles, PBS (200μL, 1x) was added to the exosome-rich supernatant, followed by brief centrifugation at 12,000 g for 2 min at 4 °C. This step facilitated the precipitation of exosome particles, with the resulting supernatant containing a higher concentration of exosomes. The harvested exosome particles were then transferred into the upper chamber of an exosome purification filter (EPF column). Subsequent centrifugation at 3,000 g and 4 °C for 10 min facilitated the separation of purified exosome particles. The pellet collected at the bottom of the EPF column tube represented the purified exosome fraction, free from contaminants and other non-exosomal components. Finally, the purified exosomes were aliquoted into volumes of 50-100μL and stored at -80 °C to maintain their stability and integrity for future experimental analyses. This meticulous isolation protocol ensures the acquisition of highly purified exosome samples suitable for downstream applications, providing valuable insights into their functional roles and biomolecular contents in various biological contexts (Liu et al. 2021a).

### Imaging of the exosomes by Transmission Electron Microscope (TEM)

Exosomes were resuspended in 50 µl of 2% paraformaldehyde. 5 µl of exosome suspension was placed on Formvar-carbon grids (two or three grids for each exosome were prepared). 100 µl of PBS was put on a parafilm sheet for washing the grids (kept the grids wet on the side of membrane but dried on the opposite side). Grids were transferred to a 50 µl drop of 1% glutaraldehyde for 5 min and then transferred to 100 µl of ddH_2_O for 2 min. These steps were repeated seven times.

The grids were transferred from the above step to 50 µl of uranyl-oxalate solution (pH = 7) and kept for 5 min. Subsequently, the grids were transferred to a 50 µl drop of methyl cellulose-UA for 10 min on ice. The grids were removed to stainless steel loops and blotted excess fluid by filter paper with drying 5 to 10 min in the air. After drying, the grids were stored in a storage box and images were captured under the electron microscope at 80 kV (Thery et al. 2006) (Frasergen Bioinformatics Co., Ltd (Wuhan, China).

### Protein quantification by bicinchoninic acid (BCA)

BCA1-1KT kit (Sigma, USA) was used for protein quantification assay. Lysate (Umibio, no. UR33101) was added to purified extracted exosomes and kept on ice for 30 min. The volume ratio of reagent A/reagent B equalled to 50 was prepared. The total volume was calculated as 200 µl for each well multiplied by the number of total samples including standard and test samples. The standard dilution method was adopted by adding deionized water (180 µl in tube 1). Twenty-five microliters of standard and test samples were added into 96-well plates with 200 µl BCA solution and mixed moderately. Plate was covered with aluminum foil and incubated at 37℃ for 30 min. The sample was analyzed by spectrophotometer and protein concentration was calculated from the standard curve (Pandey and Mann 2000).

### Western blot for identification of exosome via marker proteins

Exosome protein concentration was mixed with 1/4 volume of 5 × loading buffer and heated for 10 min. Fifteen microliters of each sample were loaded on 8% SDS–PAGE gels and run for 90 min. Protein was transferred onto PVDF membranes and probed with CD63 (1:1000 dilutions; Abcam company, no. ab193349) and TSG101 (1:1000 dilutions; Abcam company, no. ab83) primary antibodies and developed by secondary antibody, HRP reagent (Millipore, USA) (Zhu et al. 2021).

### RNA isolation and small RNA library preparation and sequencing

Total RNA from the hippocampus tissue of each group were extracted. The trizol (Invitrogen, no.15596–026) method was used for RNA extraction and separated by 1.2% agarose gel electrophoresis. Quality was tested by using the NanoPhotometer® spectrophotometer (IMPLEN, CA, USA). Nanodrop detected the RNA purity (OD 260/280), concentration, and nucleic acid absorption peak was normal. 1 µg of total RNA was used as input for the Truseq Small RNA sample prep kit (Illumina) by following the manufacturer’s recommendations and instructions (Salomon et al. 2017). The library was purified using 6% Novex TBE PAGE gel and quantified using Picogreen on the TBS-380 Fluorometer (Turner Biosystems). miRTarBase (Huang et al. 2020) software was used to predict and analyze target genes for known and novel miRNA (John et al. 2004). Differentially expressed miRNAs target genes between WT vs ADNS and ADNS vs ADF were analyzed and performed gene ontology (GO) and KEGG enrichment were analyzed (Ashburner et al. 2000).

### Statistical Analysis

The SPSS software (International Business Machines Corporation, IBM, USA) was used for statistical analysis. All data was expressed as means ± SEM. Differences among multiple groups were analyzed by one-way analysis of variance (ANOVA) while differences between two groups were analyzed using Dunnett tests. A value of *p* < 0.05 was considered statistically significant.

## Results

### Y-Maze spontaneous alternation test showed fasudil protects memory ability of AD mice

**Fig. 1:**
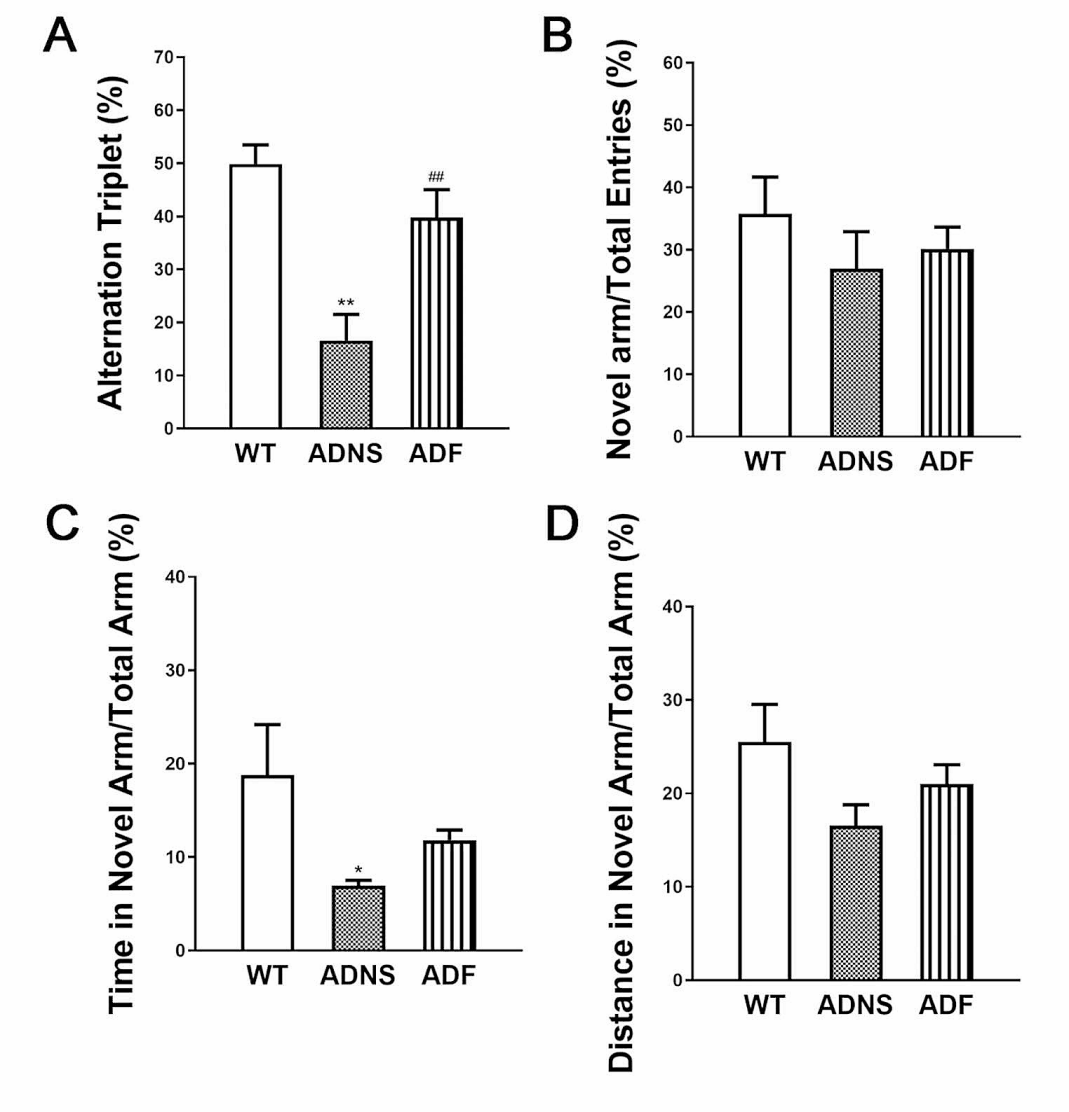
Fasudil improved the short-term memory of APP/PS1 mice in the Y maze test. **A** The percentage of spontaneous alternation rate (alternation triplet) was significantly decreased in ADNS and restored to a normal level in ADF mice. **B** The number entry in novel arm to total number of entries did not show a significant change in ADNS as compared to control WT and the number entry in novel arm to total number of entries did not show a significant change in ADNS as compared to ADF. **C** Time in novel arm to total time (%) was significantly decreased in ADNS. ADF mice showed a trend of improvement but did not show significant difference. **D** Distance travelled in novel arm to all arms (%) did not show a significant change in ANDS and ADF as compared to control WT. Data presented are the means ± SEM; *n* = 7; *p < 0.05, **p < 0.01 vs. WT; ##*p* < 0.01 vs. ADNS

Y maze test measures the dynamic spatial working memory and willingness of rodents to explore new environments. The animals tend to explore a new arm of the maze rather than returning to one that was previously visited. The total number of entries, time and distance in the new arm indicate the exploratory behavior. Alternation rate (%) reflects the memory retention. ADNS showed a significantly lower alteration rate as compared to WT (*p* < 0.01), and ADF partly restored to WT level (*p* < 0.01), suggesting that ADF treatment significantly improved the memory deficit (Fig. 1A). The ratio of the number of novel arm entry to all arms entries did not show a significant difference between all the three groups (Fig. 1B). The time spent in novel arm to all arms was significantly lower in ADNS group as compared to WT group (*p* < 0.05), and increase in ADF, but the difference was not significant (Fig. 1C). Overall, ADNS group had significantly less exploration in the novel environment as compared to WT (*p* < 0.05), and ADF improved their exploratory behavior. The ratio of distance travelled in the novel arm to all arms in ADNS group was lower as compared to WT group and increased in ADF as compared to ADNS but did not show statistically significant difference (Fig. 1D).

### Fasudil treatment altered the expression of Aβ and p-Tau in the hippocampus of AD mice

**Fig. 2:**
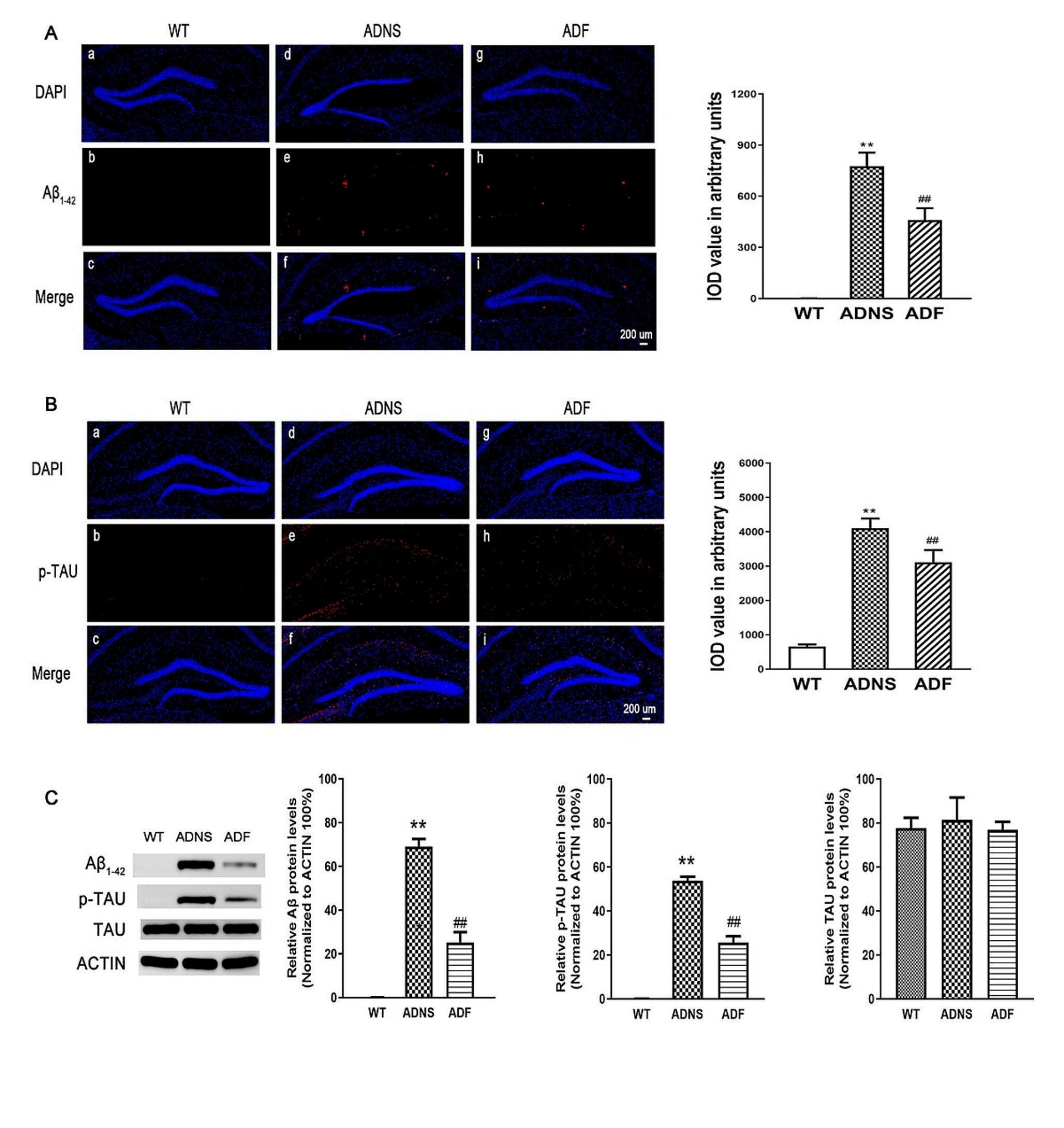
Fasudil treatment inhibited Aβ plaques deposition and phosphorylated tau tangles in the hippocampus. **A** Aβ_1-42_ plaques were checked in the hippocampus of the mice in each group. (a–c) WT, (d–f) ADNS, (g–i) ADF. Immunofluorescent intensity quantification showed a significant increased intensity of anti-Aβ1-42 in ADNS group as compared with WT and significantly decreased after fasudil-treated mice. **B** Tau phosphorylation in mice hippocampus was analyzed. (a–c) WT group, (d–f) ADNS group, (g-i) ADF group. Immunofluorescent intensity quantification of anti-Phospho-Tau showed significant increase in the ADNS group as compared to WT (*p* < 0.01) and fasudil treatment showed a significantly decreased intensity as compared to ADNS. Images (a–c, d–f, g–i) were taken at 10 × (200 μm). The box area is enlarged, and the images (d, h, l) were taken at 60 × (20 μm). DAPI (blue) was used for nuclei staining. **C** WB result of Aβ and phosphorylated TAU. Data is presented as Mean ± S.E.M. **p < 0.01 versus WT, ##*p* < 0.01 versus ADNS. The Dunnett’s test was used for statistical analysis

Expression of Aβ and p-Tau in hippocampus of the three groups (WT, ADNS, ADF) was performed by immunostaining. Aβ_1-42_ plaques in the hippocampus were significantly increased in ADNS group (776.49) and significantly decreased in ADF (459.39) (*p* < 0.01), suggesting a reduction of Aβ burden after fasudil treatment (Fig. 2A). Tau phosphorylation is a key factor for AD pathogenesis (Ando et al. 2016). Immunofluorescent intensity quantification of phospho-Tau (S404) in hippocampus was significantly increased in ADNS as compared to WT (4104.15) and decreased after fasudil treatment (3109.68) (*p* < 0.01), suggesting a protective effect of fasudil by decreasing Tau formation in AD (Fig. 2B). Western blot results showed same trend in Fig. 2C. Whole western blot picture is shown in supplementary Fig. 1.

### Exosomes extraction and identification in serum of mice

Exosomes from the serum of mice from the group were isolated using an exosome Isolation and Purification Kit (from plasma or serum) (Umibio, China) following manufacturer’s recommendations. NTA (Nanoparticle Tracking Analysis, NTA) measurements were recorded and analyzed at 11 positions. The ZetaView system was calibrated by using 110 nm polystyrene particles and the temperature was maintained between 23 °C and 30 °C.

**Fig. 3:**
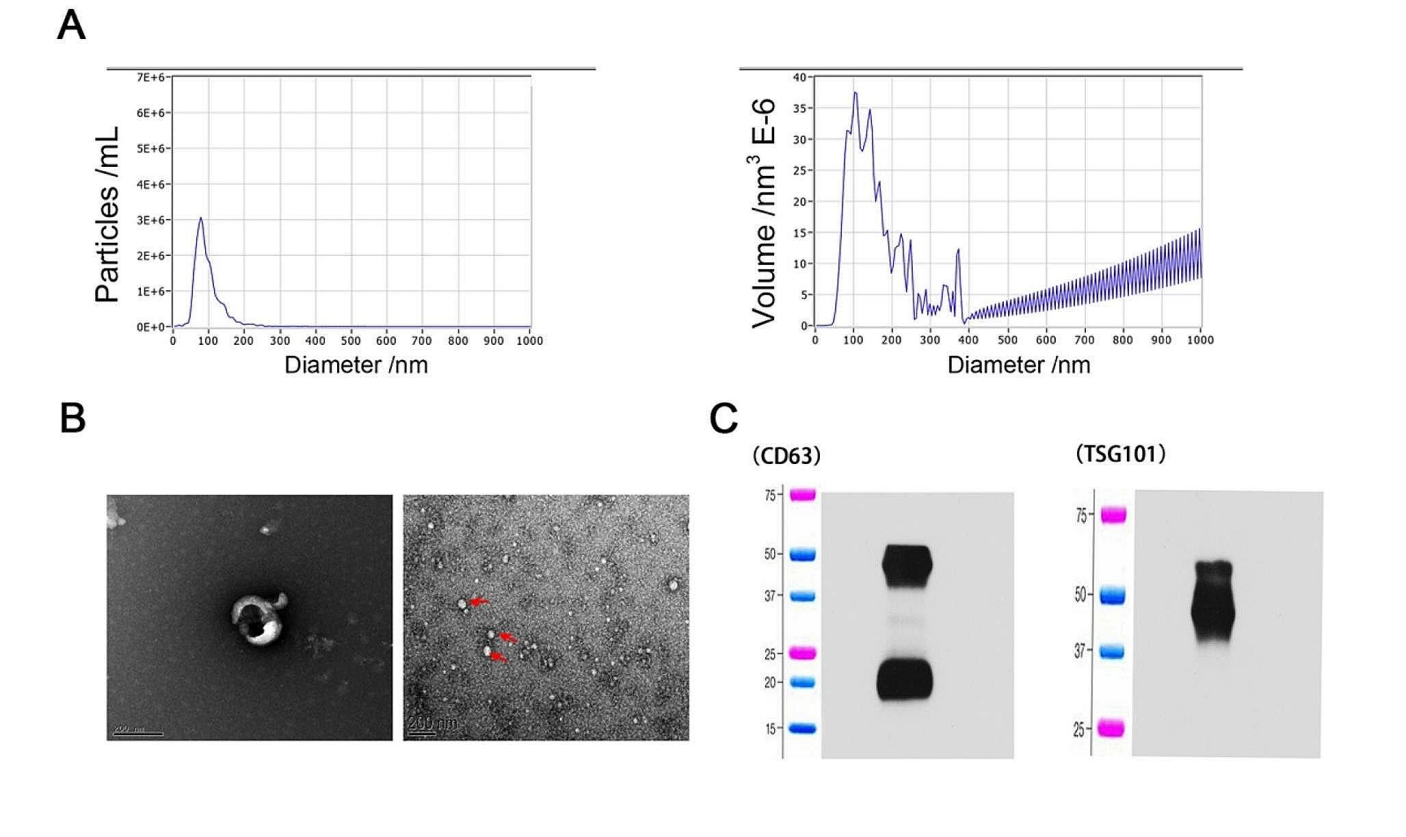
Optimization of exosomes. Exosomes were isolated from mice serum and transmission electron microscopy represent close-up images of single exosome. Nanoparticle tracking analysis provides an overview of size distribution and concentration of isolated exosomes. **A** The predominant size of exosomes was 97 nm. **B** A typical cup-shaped morphology of exosomes was observed. **C** Western blot analysis confirmed exosome by expressing their markers such as CD63 and TSG101

Transmission electron microscopy represents close-up images of a single exosome. Nanoparticle tracking analysis provides an overview of the size distribution and concentration of isolated exosomes. We observed a population of nanovesicles with a predominant size of 97 nm (Fig. 3A) which is commonly predicted the size of the exosome. A cup-shaped morphology of exosomes was also observed (Fig. 3B). Western blotting analysis confirmed that the isolated particles expressed characteristics of exosome markers of CD63 and TSG101 (Fig. 3C).

### MicroRNA profiling establishment

Exosomes of 3 mice were pooled together for sequencing as WT1 and other three mice were pooled as WT2. Similarly, we followed for ADNS1, ADNS2; ADF1 and ADF2. An unbiased high-throughput sequencing of serum exosome miRNAs was performed to capture the complete profile of miRNAs. RNA extraction from each sample yielded the typical RNA profile for exosomes, without ribosomal RNA and enriched small RNA species (< 200 nt). The expression profiles of exosomal miRNAs were analyzed and determined by deep sequencing.

A total of 1924 miRNAs were screened in each sample. Each sample achieved miRNA read counts > 10,000,000 and the error rate was around 0.09%-0.14%. Read counts were normalized to adjust the RNA and sample-level biases. Poor-quality read was detected if the number of bases (< = 20nt) in reads exceeds 50% of the read length and the content of N in reading exceeds 10% of the length of the read. Reads with abnormal final length were removed (supplementary Table 1).

**Fig. 4:**
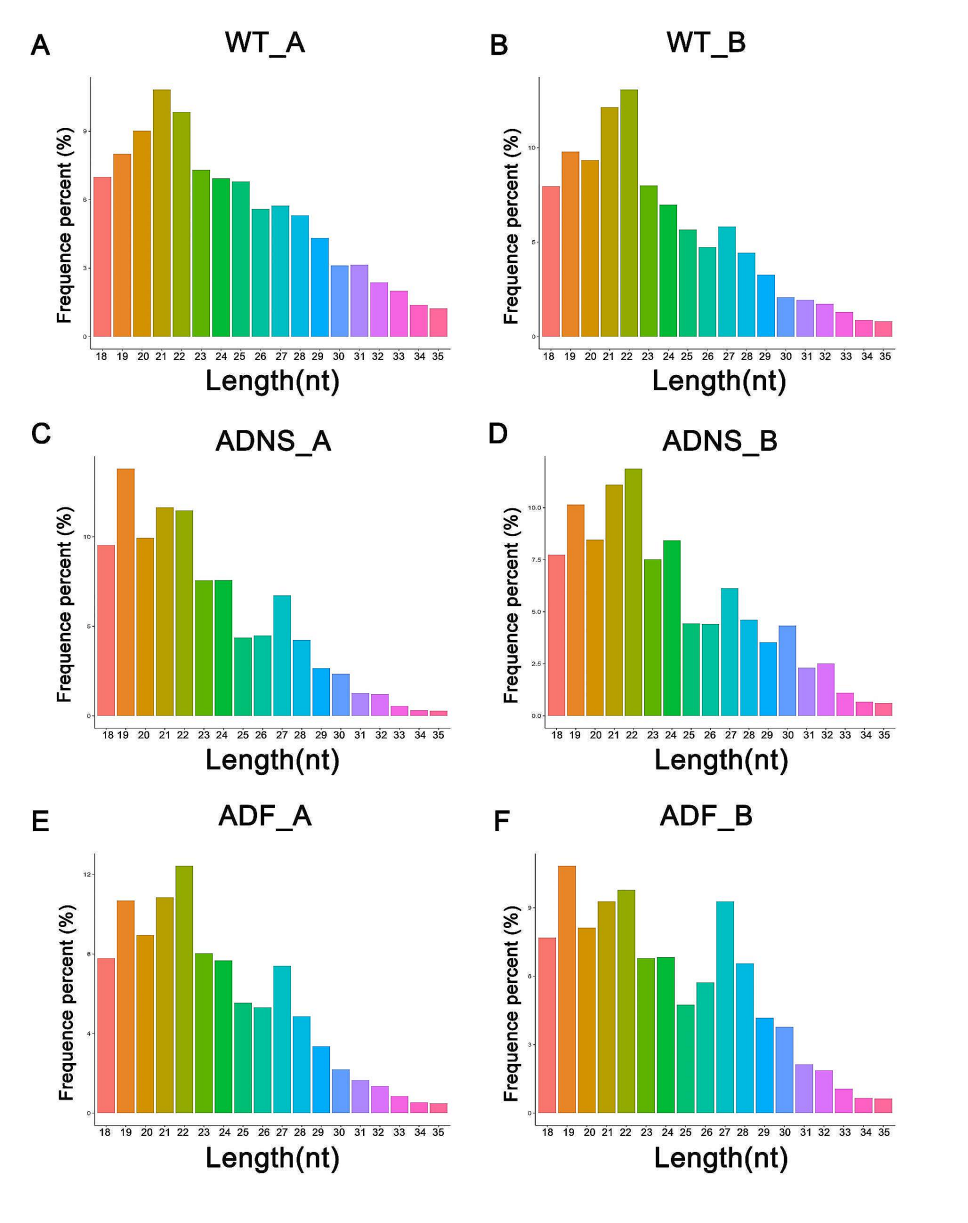
MicroRNA profile standardization. Duplicate samples (3 mice exosome pooled together in each sample) of (**A**, **B**) control (WT_A, WT_B), (**C**, **D)** AD mice treated with saline (ADNS_A, ADNS_B) and (**E–F**) AD mice treated with fasudil (ADF_A, ADF_B) were used for high-throughput sequencing of serum exosome miRNAs. Small RNA (sRNA) in the 19–22 nt length range were screened

Small RNA (sRNA) in the 19–22 nt length range were screened from clean reads of each sample for comparison and analyzed using a reference genome. These small RNA were obtained according to the priority order such as miRNA > rRNA > tRNA > snRNA > snoRNA > repeat > novel miRNA > exon > intron. Priority order of detection results were annotated for small RNAs (Fig. 4, supplementary Table 2).

### Serum exosome MicroRNA

Differential miRNA was screened, based on fold change and significant level (p/q value). Default screening conditions for differential miRNA using DESEQ2 differential analysis were *P* < 0.05 andlog_2_(fold change) > 1. We first determined whether the miRNA profile of ADNS and ADF was distinct as compared to WT by analysis of differentially expressed miRNAs.

**Fig. 5:**
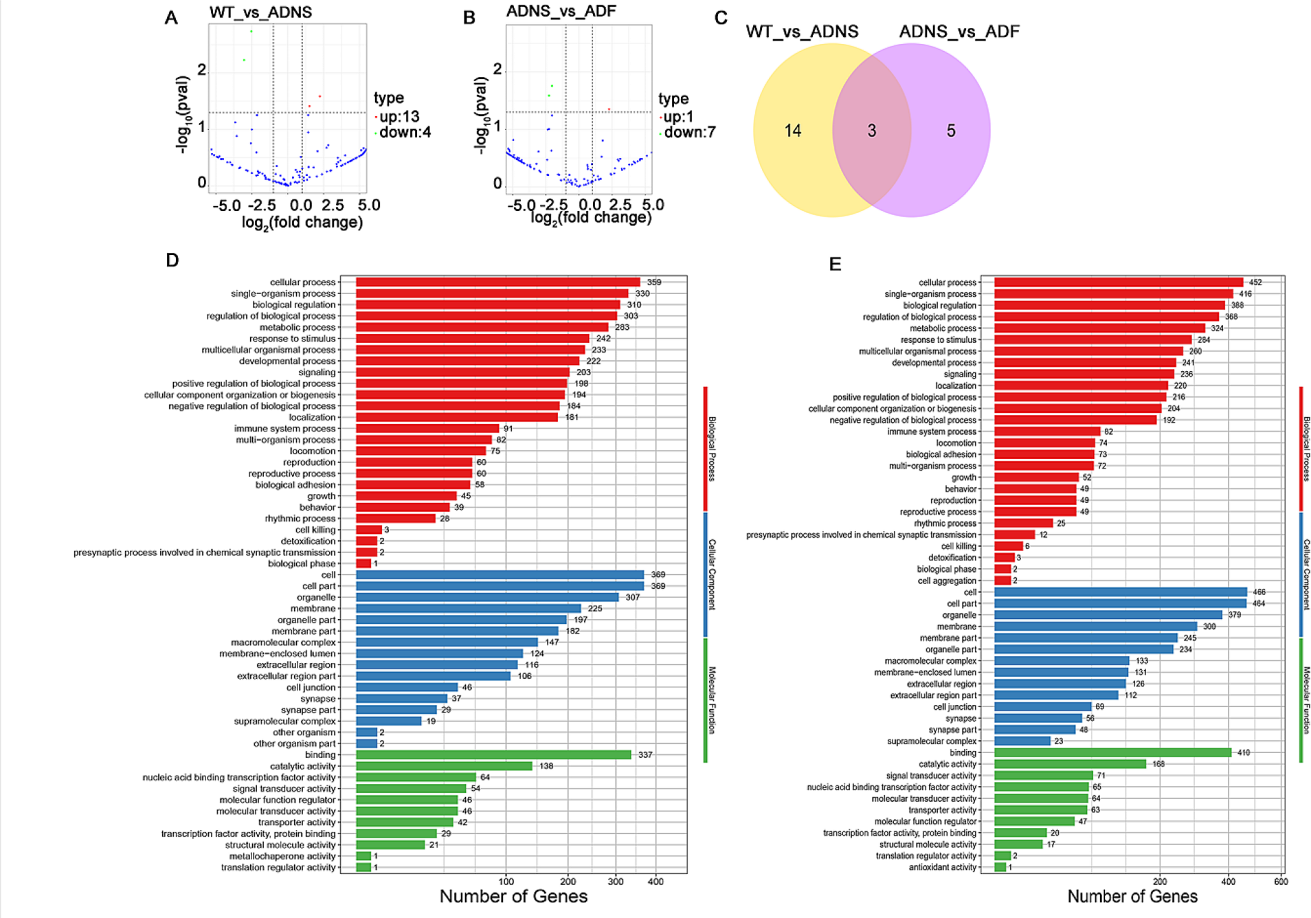
Differentially expressed serum exosome MicroRNA: **A** There were 17 significantly differentially expressed miRNAs, thirteen upregulated and four downregulated MicroRNA in ADNS and WT. **B** There were 8 significantly differentially expressed miRNAs, one upregulated and seven downregulated MicroRNA in ADNS and ADF. **C** Venn diagram showed 3 miRNAs at the intersection of ADNS versus ADF and WT versus ADNS. Gene ontology (GO) enrichment analysis of differentially expressed miRNAs target genes in (**D**) WT vs ADNS and (**E**) ADNS vs ADF showed important biological processes (cellular process, single-organism process, biological regulation, regulation of biological process), cellular components (cell, cell part, organelle, membrane) and the molecular functions (binding, catalytic activity, nucleic acid binding transcription factor activity, signal and molecular transducer activity)

There were 17 significantly differentially expressed miRNAs, including 13 up-regulated (mmu-miR-25-3p, mmu-miR-144-3p, novel_131, mmu-miR-126a-3p, mmu-miR-142a-3p, mmu-miR-142b, mmu-miR-140-3p, mmu-miR-451a, mmu-let-7i-5p, mmu-miR-1a-3p, mmu-miR-1b-5p, mmu-miR-16-5p, mmu-miR-19a-3p, 76.5%) and 4 down-regulated (novel_130, novel_56, mmu-miR-1903, mmu-miR-7058-3p, 23.5%) in ADNS compare with WT (Fig. 5A, supplementary Table 3). There were 8 significantly differentially expressed miRNAs (DEMs), including 1 up-regulated (12.5%) (mmu-let-7i-5p,) and 7 (87.5%) down-regulated mmu-miR-130b-3p, novel_63, mmu-miR-19a-3p, mmu-miR-451a, mmu-miR-423-5p, mmu-miR-574-5p, mmu-miR-466i-5p) in ADF as compared to ADNS (Fig. 5B, supplementary Table 3). There were 3 miRNAs in the intersection of ADNS vs ADF and WT vs ADNS (mmu-let-7i-5p, mmu-miR-19a-3p, mmu-miR-451a). let-7i-5p expression was significantly higher in ADF as compare with ADNS and WT. mmu-miR-19a-3p and mmu-miR-451a expression in ADNS was significantly higher than WT, ADF (Fig. 5C, supplementary Table 3).

### Serum exosome MicroRNA target genes and pathway analysis

We used MiRanda (Zhou et al. 2018) software and qTar software to predict and analyze target genes for these known miRNAs and novel miRNA targeting mmu-let-7i-5p, mmu-miR-19a-3p and mmu-miR-451a (supplementary Table 4).

#### Serum exosome MicroRNA target genes (GO)

Biological functions of differentially expressed miRNAs target genes in WT vs ADNS or ADNS vs ADF were analyzed by gene ontology (GO) enrichment analysis. GO terms showed important biological processes such as cellular process, single-organism process, biological regulation, and regulation of biological process; the most important cellular components involved the cell, cell part, organelle, membrane; and the molecular functions include binding, catalytic activity, nucleic acid binding transcription factor activity, signal and molecular transducer activity (Fig. 5D and E).

#### Serum exosome MicroRNA target genes (KEGG)

**Fig. 6:**
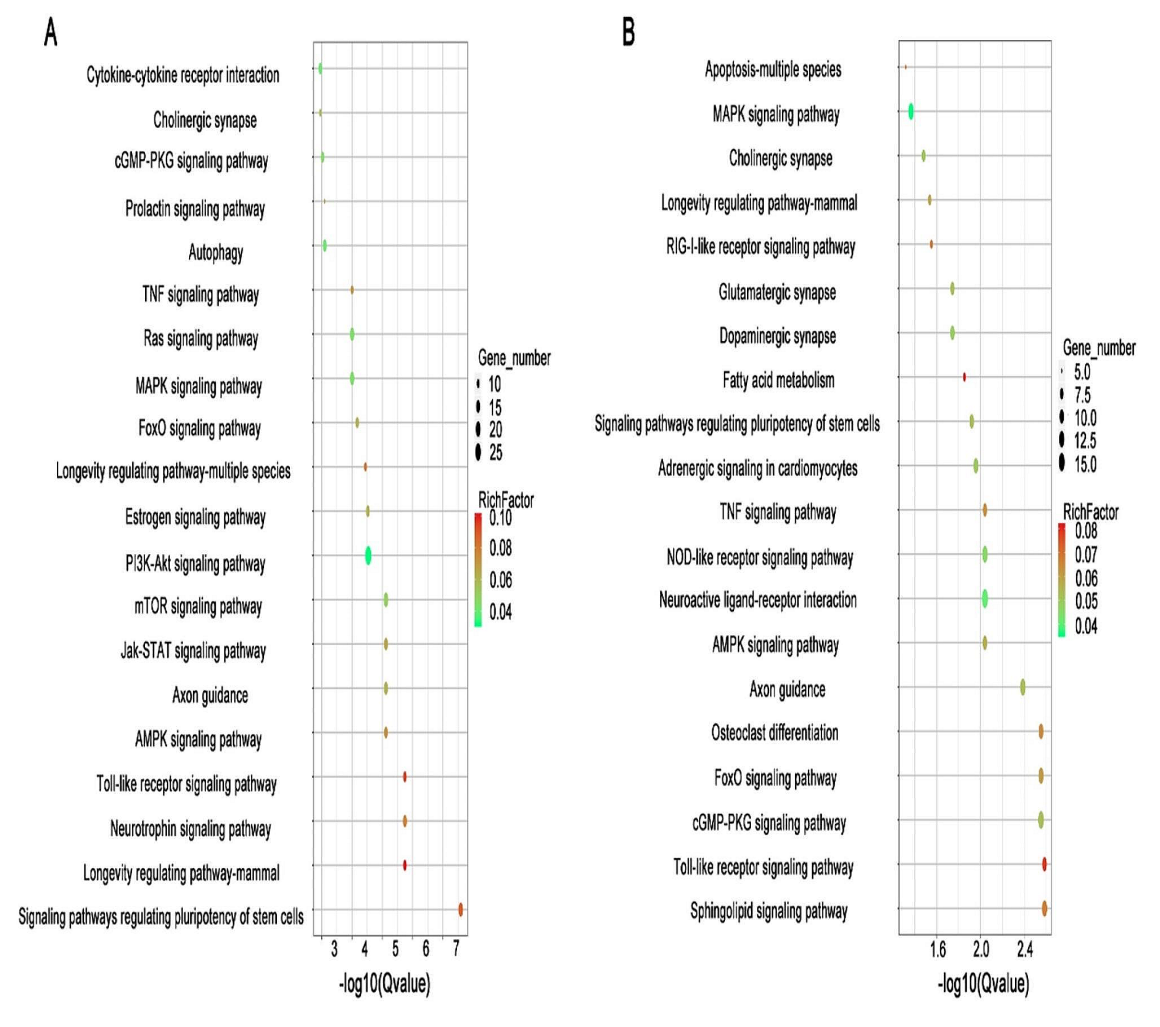
KEGG analysis of exosome MicroRNA: **A** KEGG analysis of differentially expressed miRNAs target genes between WT vs ADNS involved 181 pathways. It includes signal transduction pathways such (AMPK, Jak-STAT, mTOR, PI3K-Akt, FoxO, MAPK, Ras, TNF and cGMP-PKG), neurotrophin signaling pathway, cholinergic synapse, endocrine system (estrogen signaling pathway) and others (autophagy, axon guidance, cytokine-cytokine receptor interaction and signaling pathways regulating pluripotency of stem cells). **B** The miRNAs target genes of ADNS vs ADF showed 195 pathways. It includes signal transduction (sphingolipid, cGMP—PKG, FoxO, AMPK, TNF and MAPK signaling), immune system (Toll-like receptor, NOD-like receptor and RIG-I-like receptor), developmental (osteoclast differentiation, axon guidance), nervous system (dopaminergic, glutamatergic and cholinergic synapse) and others (neuroactive ligand-receptor interaction, adrenergic signaling, pluripotency of stem cells, fatty acid metabolism, longevity regulating pathway and apoptosis) (KEGG pathways: https://www.kegg.jp/kegg/kegg1.html)

Differentially expressed miRNAs target genes between WT vs ADNS involved 181 pathways. Signal transduction includes AMPK, Jak-STAT, mTOR, PI3K-Akt, FoxO, MAPK, Ras, TNF and cGMP-PKG signaling pathways. Aging includes longevity regulating pathways in the nervous system such as the neurotrophin signaling pathway and Cholinergic synapse. The endocrine system includes estrogen and prolactin signaling pathways. Other pathways are autophagy, axon guidance, cytokine-cytokine receptor interaction and signaling pathways regulating pluripotency of stem cells (Fig. 6).

Differentially expressed miRNAs target genes between ADNS vs ADF showed 195 pathways. Signal transduction includes sphingolipid, cGMP - PKG, FoxO, AMPK, TNF and MAPK signaling pathway. The immune system includes a Toll-like receptor, a NOD-like receptor and a RIG-I-like receptor signaling pathway. Developmental includes osteoclast differentiation and axon guidance. The nervous system includes dopaminergic, glutamatergic and cholinergic synapses. Other pathways were neuroactive ligand-receptor interaction, adrenergic signaling in cardiomyocytes, signaling pathways regulating pluripotency of stem cells, fatty acid metabolism, longevity regulating pathway and apoptosis (Fig. 6)**.**

#### Exosomal MicroRNA target genes (KEGG) in the intersection of ADNS vs ADF and WT vs ADNS

mmu-miR-19a-3p and mmu-miR-451a were in the intersection of ADNS vs ADF and WT vs ADNS and were significantly higher in ADNS as compared to WT and ADF.

##### mmu-miR-19a-3p

The target genes of mmu-miR-19a-3p were involved in 44 pathways, including 12 major signal pathways such as signal transduction (Sphingolipid, FoxO and TNF signaling pathway), immune system (Toll-like receptor signaling pathway), signaling molecules and interaction (neuroactive ligand-receptor interaction), cellular community (Signaling pathways regulating pluripotency of stem cell) and nervous system (Cholinergic synapse).

The phosphatase and tensin homolog (Pten, ENSMUSG00000013663) is one of the target genes of mmu-miR-19a-3p which is associated with the sphingolipid and FoxO signaling pathway. Another target gene of mmu-miR-19a-3p is tumor necrosis factor (Tnf, ENSMUSG00000024401) which is involved in signal transduction pathways such as sphingolipid, TNF, immune system and Toll-like receptor signaling pathways.

##### mmu-miR-451a

The target genes of mmu-miR-451a involved 89 pathways. Significantly involved were 19 pathways such as signal transduction (sphingolipid, cGMP-PKG, FoxO and TNF signaling pathway), immune system (Toll-like receptor signaling pathway), signaling molecules and interaction (neuroactive ligand-receptor interaction), cellular community (signaling pathways regulating pluripotency of stem cell), nervous system (glutamatergic, dopaminergic and cholinergic synapse).

The target gene of mmu-miR-451a is (1) Nsmaf (ENSMUSG00000028245, neutral sphingomyelinase activation associated factor) in the sphingolipid signaling pathway, (2) Gnai3 (G protein, alpha inhibiting 3, ENSMUSG00000000001) in the sphingolipid signaling pathway, glutamatergic synapse, dopaminergic synapse, cholinergic synapse and cGMP—PKG signaling pathway, (3) Akt3 (ENSMUSG00000019699, thymoma viral proto-oncogene 3) in sphingolipid, Toll-like receptor, FoxO, TNF signaling pathway, dopaminergic synapse, signaling pathways regulating pluripotency of stem cells, and (4) Myc (myelocytomatosis oncogene, ENSMUSG00000022346) in the signaling pathways regulating pluripotency of stem cells.

## Discussion

Our previous study showed that fasudil ameliorates cognitive function in the Morris water maze (MWM) behavior test (Yan et al. 2021). The present study also showed amelioration of cognitive function in Y maze test after fasudil treatment in APP/PS1 mice. We believe that fasudil induced cognitive improvement could be due to the manipulation of serum exosomal miRNAs, and mmu-miR-19a-3p and mmu-miR-451a, which could be potential target biomarkers of fasudil treatment in APP/PS1mice.

**Fig. 7:**
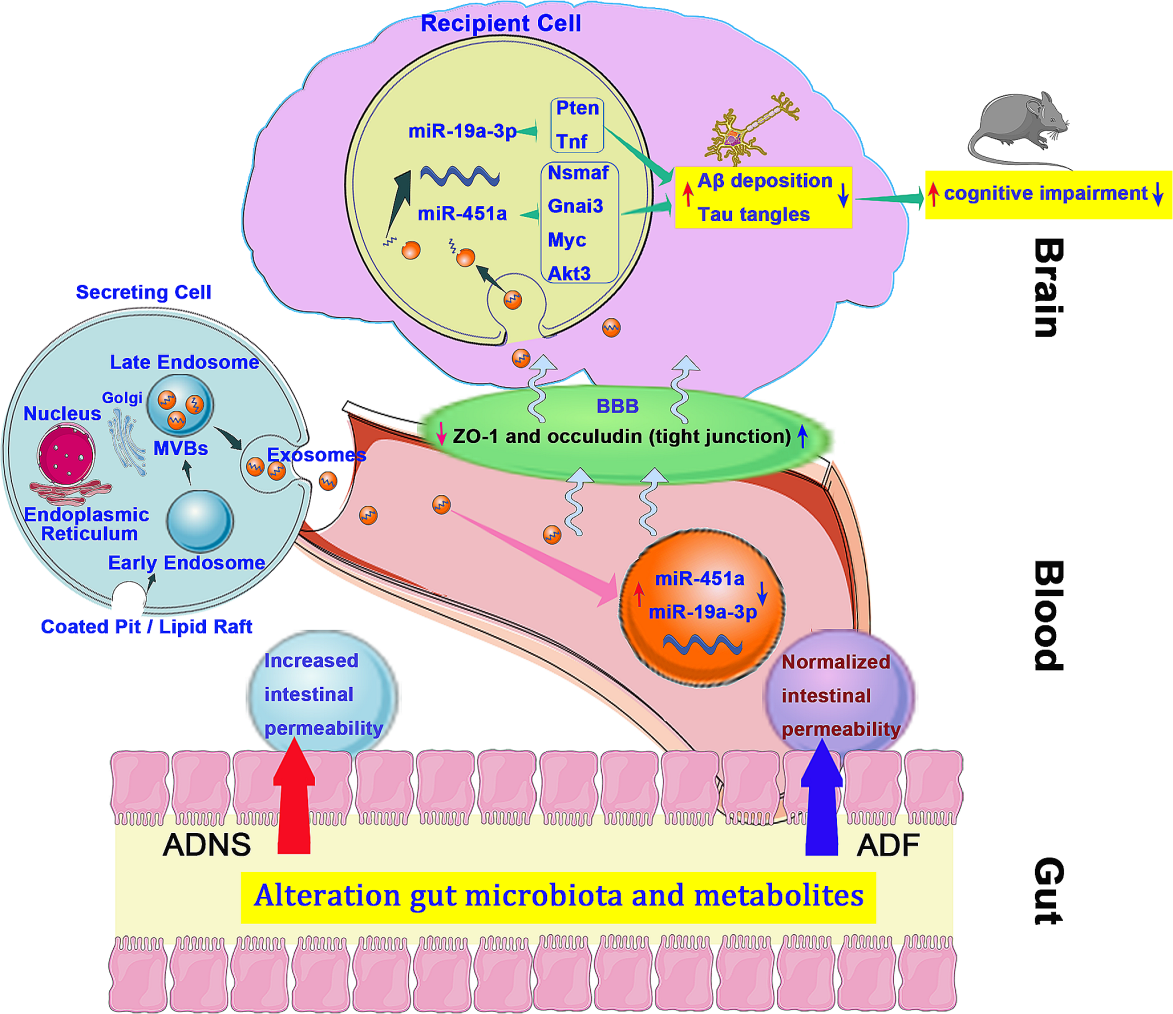
Exosomal MicroRNAs as novel target for diagnosis and treatment of AD by manipulating gut-brain axis. Gut microbes are involved in the regulation of genes and maintenance of intestinal homeostasis of host. Microbiota promotes the proliferation from intestinal epithelial stem cells (IESC). Exosomal MicroRNAs (mmu-miR-451a and mmu-miR-19a-3p) crosses the blood brain barrier in disease condition such as AD. Pten and Tnf for mmu-miR-451a and Nsmaf, Gnai3, Myc, Akt3 for mmu-miR-19a-3p are target genes orchestrates the Aβ plaque deposition and tau pathology. Fasudil treatment improved cognitive function by regulating these exosomal MicroRNAs. These MicroRNAs could be potential biomarker of AD and potential target for novel treatment for AD

In our previous and recent studies (Yan et al. 2021, 2022), we discovered that AD progression is associated with the alteration of gut microbiota and metabolites and impacts the cognitive function. There are several cellular communications in AD. One of them is exosomes that carry proteins, RNAs and DNAs and transport them from cell to cell. Our previous studies have shown the relationship between intestinal microbiota and AD through the microbiota–gut-brain axis. Intestinal microbiota and exosome miRNA could serve as new targets for AD therapeutic intervention. Exosomes are practical for long-term storage and easily pass through the blood–brain barrier (BBB) while protecting their molecules wrapped in their bilayer lipid structure (Wang et al. 2022). We believe that brain-gut axis results in the transportation of biochemical molecules between the brain and gut via exosomes (Liu et al. 2021b). Therefore, intestinal flora and exosomes carrying biochemical products could trigger neurodegenerative diseases through the brain-gut axis, which may be a potential mechanism for AD onset and a target for drug therapy (Sugiura et al. 2021). Based on the metagenome and exosome results, we will continue our study of the brain-gut axis pathway and fasudil treatment in AD to reveal the novel pathophysiology of AD and systematically elaborate the mechanism of fasudil treated AD (Fig. 7).

### mmu-miR-19a-3p

miRNA alterations in CNS and PNS influence microglial activity and neuroinflammation by orchestrating the expression of proteins, which controls the activation or inhibition of microglial activity. A recent clinical study reports that hsa-miR-19a-3p and hsa-miR-19b-3p expression distinctly discriminate the pain severity in spinal cord injury-induced neuropathic pain (Ye et al. 2021). Increased expression of miR-19a-3p in the exosomes of α-synuclein gene transgenic SH-SY5Y cells has been reported, while enhanced expression of miR-19a-3p in exosomes suppresses autophagy in recipient microglia by targeting the phosphatase and tensin homolog/AKT/mTOR signaling pathway (Zhou et al. 2019). In multiple systems atrophy (MSA), miRNA expression and corresponding gene target suggest that miR-19a-3p acts as key regulators of neuroinflammation (Kim et al. 2019). A similar trend was observed in our study, where mmu-miR-19a-3p expression was significantly decreased after fasudil treatment. Therefore, we speculate a potential link between AD and MSA pathologies that involves miRNAs and deregulation of BACE1.

Pten is the target gene of mmu-miR-19a-3p in the sphingolipid and FoxO signaling pathways. Pten is a tumor suppressor gene regulating axonal growth in the adult central nervous system (Park et al. 2008). Pten deletion enhances neurite outgrowth during neural stem cell differentiation (Shen et al. 2020). Phosphatidylinositol-4,5-bisphosphate 3-kinase (PI3K)/protein kinase B (PKB or Akt) pathway is deregulated in response to phosphatase and tensin homolog (Pten) overexpression in AD by orchestrating inflammation and oxidative stress. AD patient’s serum has shown an increased level of amyloid β (Aβ) 42, p-tau as well as *PTEN* (Mohamed et al. 2019).

In our investigation, we found that tumor necrosis factor (TNF) is targeted by mmu-miR-19a-3p, particularly within signaling pathways such as the sphingolipid and TNF signaling pathway, as well as immune system pathways like the Toll-like receptor signaling pathway. This finding is significant, as elevated levels of TNF-α, interferon gamma (IFN-γ), and interleukin 6 (IL-6) have been consistently observed in patients with Alzheimer's disease (AD) compared to healthy controls (Iulita et al. 2016). Emerging research suggests that targeting neuroinflammation could hold promise as a therapeutic or preventive strategy for AD. By inhibiting the inflammatory responses mediated by cytokines like TNF-α, IFN-γ, and IL-6, it may be possible to mitigate the neuroinflammatory processes that contribute to AD pathology (Jiang et al. 2019; Dhapola et al. 2021).

### mmu-miR-451a

miRNA451a expression in AD patients has been reported to increase significantly as compared to healthy control and positively correlates with Aβ-42/Aβ-40 ratio and tau protein (Samadian et al. 2021; Kuang et al. 2018). We also observed significantly increased expression of miRNA451a in AD mice and restoration to a normal level after fasudil treatment.

Sphingolipid metabolism has been associated with amyloid-beta production and AD neuropathology (Haughey et al. 2010). Nsmaf is a target gene of mmu-miR-451a in the sphingolipid signaling pathway and is reported to regulate AD patient’s blood (Bai et al. 2014).

Gnai3 is a target gene of mmu-miR-451a in the sphingolipid signaling pathway, glutamatergic synapse, dopaminergic synapse, cholinergic synapse and cGMP—PKG signaling pathway. Gnai3 assist in guanyl nucleotide binding, metal ion binding, nucleotide binding, protein binding and protein domain-specific binding. Gnai3 is associated with lipid metabolism in major depression (Leslie et al. 2014) and AD pathogenesis in the mouse model (Lin et al. 2014).

cGMP-PKG signaling pathway in the hippocampus of AD mice has been shown to dysregulates circRNAs (Kelly 2018; Ricciarelli and Fedele 2018). mmu-miR-298-3p/Smoc2 signaling axis may regulate the pathophysiology of AD by affecting the cGMP-PKG signaling pathway (Zhang et al. 2021).

Akt3 is also the target gene of mmu-miR-451a in the sphingolipid, Toll-like receptor, FoxO and TNF signaling pathway along with dopaminergic synapse, signaling pathways regulating pluripotency of stem cells. Akt3 is the predominant isoform of Akt expressed in the hippocampus and is primarily affected during AD progression. Mitochondrial dysfunction and AD–like pathology have been reported in Akt3-null mice (Zhang et al. 2019). Akt3 modulates angiogenesis and orchestrates mitochondrial dynamics in the vascular endothelium by controlling autophagy and biogenesis through subcellular localization of the master regulator of nuclear mitochondrial gene expression, PGC-1α (Corum et al. 2020). Akt3 is also required for the nuclear export receptor, CRM-1 (Corum et al. 2014). MiR‐485‐3p serves as a biomarker and therapeutic target of AD via regulating neuronal cell viability and neuroinflammation by targeting Akt3 (Ye et al. 2021).

Myc is the target gene of mmu-miR-451a in the signaling pathways regulating the pluripotency of stem cells. Myc repairs brain cells in neurodegenerative disease or CNS trauma, including stroke and traumatic brain and spinal cord injury and promotes axonal growth and regeneration. It also regulates the cell cycle, metabolism, and enhances the synaptic structure to restore cognitive function (Corum et al. 2014).

Pten and Akt3 share a common FoxO signaling pathway. FoxO transcription factors control the proinflammatory pathways, affecting nervous system amyloid (Aβ) production and toxicity, leading to mitochondrial dysfunction, fostering neuronal apoptotic cell death, and accelerating the progression of degenerative disease (Broughton et al. 1989). However, FoxOs also offer a protective effect on the nervous system by promoting autophagy, reducing toxic intracellular protein accumulations and potentially limiting Aβ toxicity (Maiese 2017). FoxO proteins may provide a potential molecular target for the treatment of AD (Manolopoulos et al. 2010).

## Conclusions

The administration of fasudil has been shown to ameliorate cognitive impairments by modulating the expression of exosomal microRNAs, specifically mmu-miR-451a and mmu-miR-19a-3p. These microRNAs hold promise as potential biomarkers for Alzheimer's disease (AD) and represent viable targets for innovative therapeutic interventions. Our study not only sheds light on the pathophysiology of AD but also introduces a novel approach by targeting key signaling pathways implicated in the disease progression. By targeting signaling pathways such as signal transduction, immune system modulation, signaling molecules and interactions, cellular community dynamics, and nervous system pathways, our research aims to elucidate the intricate mechanisms underlying AD pathology. Furthermore, we investigated pathways regulating pluripotency of stem cells, recognizing their potential role in neuroregeneration and repair processes, which are crucial for AD treatment.

One of the primary challenges in AD drug development has been the lack of precise understanding of the underlying mechanisms driving the disease. Traditional drug development strategies often focus on single-target approaches, which may overlook the complex interplay of various molecular pathways involved in AD pathogenesis. To address this challenge, our approach employs a multi-target targeting strategy, aiming to intervene AD at multiple levels of disease pathology simultaneously. Moreover, our focus extends beyond symptomatic treatment to encompass preventive strategies, particularly targeting the early stages of disease development. By identifying and targeting exosomal microRNAs, which play crucial roles in AD pathogenesis, our novel approach holds significant promise for early diagnosis, intervention, and the development of new AD therapeutics. In summary, our study presents a comprehensive strategy leveraging exosomal microRNAs as potential biomarkers and therapeutic targets for AD. By elucidating the complex signaling pathways involved in AD pathology and adopting a multi-target approach, we aim to pave the way for innovative diagnostic tools and therapeutic interventions that could revolutionize the management of this devastating neurodegenerative disease.

## Supplementary Information

Below is the link to the electronic supplementary material.

Supplementary file1 (PDF 3239 kb)

Supplementary figure 1 Whole western blot picture of Aβ, Tau, phosphorylated Tau and beta actin. Whole western blot picture shows (A) β-amyloid (Aβ) (100 KDa), (B) Tau (79 KDa), (C) phosphorylated TAU (p-TAU) (79 KDa) and (D) beta actin (45 KDa). (PNG 718 kb)

High Resolution Image (TIF 8970 kb)

## Data Availability

The raw data of this paper is available in the public database (Sequence Read Achieved; SUB11804148; Ref: PRJNA859243). The data that support the findings of this paper is available in the published articles and are additional files.
