# Supplementary material for: Exosomal MicroRNAs modulate the cognitive function in fasudil treated APPswe/PSEN1dE9 transgenic (APP/PS1) mice model of Alzheimer’s disease
Source: Metab Brain Dis. 2024 Aug 1;39(7):1335–51. doi: 10.1007/s11011-024-01395-8 (PMC11513711; doi:10.1007/s11011-024-01395-8)
Supplement: Supplementary file 1 — Supplementary file1 (PDF 3239 kb) [file 11011_2024_1395_MOESM1_ESM.pdf]

Supplementary table 1

Exosomal MicroRNAs modulate the cognitive function in fasudil treated APPswe/PSEN1dE9 transgenic (APP/PS1) mice model of Alzheimer's disease

Yuqing Yan[1*#], Ye Gao[1*], Gajendra Kumar[3*#], Qingli Fang[1], Hailong Yan[1], Nianping Zhang[1], Yuna Zhang[1], Lijuan Song[2], Jiehui Li[1], Yucheng Zheng[1], Nan Zhang[1], Peijun Zhang[1], Cungen Ma[1, 2#]

1. Institute of Brain Science, Shanxi Key Laboratory of Inflammatory Neurodegenerative Diseases, Medical School of Shanxi Datong University, Datong, China.
2. The Key Research Laboratory of Benefiting Qi for Acting Blood Circulation Method to Treat Multiple Sclerosis of State Administration of Traditional Chinese Medicine, Research Center of Neurobiology, Shanxi University of Chinese Medicine, Taiyuan, China.
3. Department of Neuroscience, City University of Hong Kong, Kowloon, Hong Kong.

* Contributed equally.
# Corresponding author (Yuqing Yan: 779734216@qq.com; Gajendra Kumar: gajendra_kumar@brown.edu, , Cungen Ma: macungen@sxtcm.edu.cn).

Supplementary table 1: Sample validation using read counts and error rate.

Sample	Raw Reads	Bases	Error rate	Q20	Q30	GC_content	clean reads
WT_A	10114488	0.759G	0.09%	85.38%	74.90%	37.14%	3765627
WT_B	15649992	1.174G	0.14%	81.26%	71.29%	35.63%	3164192
ADNS_A	12170574	0.913G	0.10%	84.74%	73.66%	39.00%	3151328
ADNS_B	15584627	1.169G	0.14%	81.06%	70.73%	31.96%	2918508
ADF_A	12046496	0.903G	0.09%	84.91%	74.44%	38.65%	3587425
ADF_B	10480737	0.786G	0.09%	85.97%	75.40%	41.00%	3833080

Exosomal MicroRNAs modulate the cognitive function in fasudil treated APPswe/PSEN1dE9 transgenic (APP/PS1) mice model of Alzheimer's disease

Yuqing Yan[1*#], Ye Gao[1*], Gajendra Kumar[3*#], Qingli Fang[1], Hailong Yan[1], Nianping Zhang[1], Yuna Zhang[1], Lijuan Song[2], Jiehui Li[1], Yucheng Zheng[1], Nan Zhang[1], Peijun Zhang[1], Cungen Ma[1, 2#]

1. Institute of Brain Science, Shanxi Key Laboratory of Inflammatory Neurodegenerative Diseases, Medical School of Shanxi Datong University, Datong, China.
2. The Key Research Laboratory of Benefiting Qi for Acting Blood Circulation Method to Treat Multiple Sclerosis of State Administration of Traditional Chinese Medicine, Research Center of Neurobiology, Shanxi University of Chinese Medicine, Taiyuan, China.
3. Department of Neuroscience, City University of Hong Kong, Kowloon, Hong Kong.

* Contributed equally.
# Corresponding author (Yuqing Yan: 779734216@qq.com; Gajendra Kumar: gajendra_kumar@brown.edu, , Cungen Ma: macungen@sxtcm.edu.cn).

Supplementary table 2: Priority order of Small RNA (sRNA).

**Types**	**ADF_A**	**ADF_A (percent)**	**ADF_B**	**ADF_B (percent)**	**ADNS_A**	**ADNS_A (percent)**	**ADNS_B**	**ADNS_B (percent)**	**WT_A**	**WT_A (percent)**	**WT_B**	**WT_B (percent)**
total	2734952	100%	3008437	100%	2817263	100%	2606403	100%	3309916	100%	2630666	100%
known_miRNA	5631	0%	6721	0%	7077	0%	5565	0%	5235	0%	5358	0%
rRNA	1643733	60%	2019593	67%	2058486	73%	1937039	74%	989965	30%	1187153	45%
tRNA	10510	0%	11156	0%	10988	0%	10809	0%	10796	0%	16149	1%
snRNA	78310	3%	66380	2%	92660	3%	68786	3%	228357	7%	120080	5%
snoRNA	12926	0%	7089	0%	8572	0%	7824	0%	30872	1%	17806	1%
repeat	513503	19%	389576	13%	270555	10%	246434	9%	1486776	45%	794220	30%
novel_miRNA	955	0%	432	0%	688	0%	396	0%	701	0%	885	0%
exon	66573	2%	45438	2%	44744	2%	32146	1%	147210	4%	114127	4%
intron	49955	2%	39118	1%	29890	1%	30317	1%	121697	4%	88806	3%
other	352856	13%	422934	14%	293603	10%	267087	10%	288307	9%	286082	11%

Exosomal MicroRNAs modulate the cognitive function in fasudil treated APPswe/PSEN1dE9 transgenic (APP/PS1) mice model of Alzheimer's disease

Yuqing Yan[1*#], Ye Gao[1*], Gajendra Kumar[3*#], Qingli Fang[1], Hailong Yan[1], Nianping Zhang[1], Yuna Zhang[1], Lijuan Song[2], Jiehui Li[1], Yucheng Zheng[1], Nan Zhang[1], Peijun Zhang[1], Cungen Ma[1, 2#]

1. Institute of Brain Science, Shanxi Key Laboratory of Inflammatory Neurodegenerative Diseases, Medical School of Shanxi Datong University, Datong, China.
2. The Key Research Laboratory of Benefiting Qi for Acting Blood Circulation Method to Treat Multiple Sclerosis of State Administration of Traditional Chinese Medicine, Research Center of Neurobiology, Shanxi University of Chinese Medicine, Taiyuan, China.
3. Department of Neuroscience, City University of Hong Kong, Kowloon, Hong Kong.

* Contributed equally.
# Corresponding author (Yuqing Yan: 779734216@qq.com; Gajendra Kumar: gajendra_kumar@brown.edu, , Cungen Ma: macungen@sxtcm.edu.cn).

Supplementary table 3a: Differential miRNA was screened, based on fold change and significant level (p/q value) for WT_vs_ADNS.

**sRNA_tpm**	**WT_A**	**WT_B**	**ADNS_A**	**ADNS_B**	**log2FoldChange**	**P value**
novel_130	39462.86654	0	0	0	-9.5033	0.047063
novel_56	5755.00137	7638.732869	0	0	-7.448	0.0066963
mmu-miR-1903	25760.48232	14378.79128	4415.011038	1239.157373	-3.04	0.0058736
mmu-miR-7058-3p	58098.10907	40889.68771	7910.228109	11648.07931	-2.5302	0.0018204
mmu-miR-25-3p	24116.19622	36845.65266	53532.00883	120693.9281	1.512	0.038549
mmu-miR-144-3p	2192.381474	7638.732869	14900.66225	26517.96778	2.2458	0.025886
novel_131	0	224.6686138	2391.464312	10408.92193	5.7948	0.0283
mmu-miR-126a-3p	0	0	5334.805004	2230.483271	6.3615	0.048557
mmu-miR-142a-3p	0	0	5150.84621	3221.80917	6.505	0.037973
mmu-miR-142b	0	0	5150.84621	3221.80917	6.505	0.037973
mmu-miR-140-3p	0	0	4047.093451	4708.798017	6.5648	0.034056
mmu-miR-451a	0	0	3863.134658	5204.460967	6.6142	0.031333
mmu-let-7i-5p	0	0	2391.464312	6939.281289	6.6503	0.030465
mmu-miR-1a-3p	0	0	10853.5688	247.8314746	6.9224	0.026137
mmu-miR-1b-5p	0	0	10853.5688	247.8314746	6.9224	0.026137
mmu-miR-16-5p	0	0	919.7939662	12887.23668	7.2102	0.011791
mmu-miR-19a-3p	0	0	2207.505519	14374.22553	7.4763	0.0047744

Exosomal MicroRNAs modulate the cognitive function in fasudil treated APPswe/PSEN1dE9 transgenic (APP/PS1) mice model of Alzheimer's disease

Yuqing Yan[1*#], Ye Gao[1*], Gajendra Kumar[3*#], Qingli Fang[1], Hailong Yan[1], Nianping Zhang[1], Yuna Zhang[1], Lijuan Song[2], Jiehui Li[1], Yucheng Zheng[1], Nan Zhang[1], Peijun Zhang[1], Cungen Ma[1, 2#]

1. Institute of Brain Science, Shanxi Key Laboratory of Inflammatory Neurodegenerative Diseases, Medical School of Shanxi Datong University, Datong, China.
2. The Key Research Laboratory of Benefiting Qi for Acting Blood Circulation Method to Treat Multiple Sclerosis of State Administration of Traditional Chinese Medicine, Research Center of Neurobiology, Shanxi University of Chinese Medicine, Taiyuan, China.
3. Department of Neuroscience, City University of Hong Kong, Kowloon, Hong Kong.

* Contributed equally.
# Corresponding author (Yuqing Yan: 779734216@qq.com; Gajendra Kumar: gajendra_kumar@brown.edu, , Cungen Ma: macungen@sxtcm.edu.cn).

Supplementary table 3b: Differential miRNA was screened, based on fold change and significant level (p/q value) for ADNS_vs_ADF.

**sRNA_tpm**	**ADNS_A**	**ADNS_B**	**ADF_A**	**ADF_B**	**log2FoldChange**	**pval**
**mmu-miR-130b-3p**	5334.805004	15613.3829	0	0	-7.8612	0.0020358
**novel_63**	9197.939662	5700.123916	0		-7.5538	0.0062166
**mmu-miR-19a-3p**	2207.505519	14374.22553	0	0	-7.4565	0.0092767
**mmu-miR-451a**	3863.134658	5204.460967	0	0	-6.7432	0.049054
**mmu-miR-423-5p**	7726.269316	8426.270136	258.8661662	0	-6.1866	0.015919
**mmu-miR-574-5p**	36975.71744	19578.68649	10354.64665	5743.243243	-2.2644	0.025623
**mmu-miR-466i-5p**	310154.5254	158116.4808	105099.6635	51013.51351	-2.0394	0.017422
**mmu-let-7i-5p**	2391.464312	6939.281289	24851.15195	29391.89189	2.2615	0.044319

Exosomal MicroRNAs modulate the cognitive function in fasudil treated APPswe/PSEN1dE9 transgenic (APP/PS1) mice model of Alzheimer's disease

Yuqing Yan[1*#], Ye Gao[1*], Gajendra Kumar[3*#], Qingli Fang[1], Hailong Yan[1], Nianping Zhang[1], Yuna Zhang[1], Lijuan Song[2], Jiehui Li[1], Yucheng Zheng[1], Nan Zhang[1], Peijun Zhang[1], Cungen Ma[1, 2#]

1. Institute of Brain Science, Shanxi Key Laboratory of Inflammatory Neurodegenerative Diseases, Medical School of Shanxi Datong University, Datong, China.
2. The Key Research Laboratory of Benefiting Qi for Acting Blood Circulation Method to Treat Multiple Sclerosis of State Administration of Traditional Chinese Medicine, Research Center of Neurobiology, Shanxi University of Chinese Medicine, Taiyuan, China.
3. Department of Neuroscience, City University of Hong Kong, Kowloon, Hong Kong.

* Contributed equally.
# Corresponding author (Yuqing Yan: 779734216@qq.com; Gajendra Kumar: gajendra_kumar@brown.edu, , Cungen Ma: macungen@sxtcm.edu.cn).

Supplementary table 3c: Differential miRNA for WT_vs_ADNS and ADNS_vs_ADF.venn.

	WT_vs_ADNS							ADNS_vs_ADF					
**sRNA_tpm**	**WT_A**	**WT_B**	**ADNS_A**	**ADNS_B**	**log2FoldChange**	**pval**		**ADNS_A**	**ADNS_B**	**ADF_A**	**ADF_B**	**log2FoldChange**	**P value**
**mmu-let-7i-5p**	0	0	2391.464312	6939.281289	6.6503	0.030465		2391.464312	6939.281289	24851.15195	29391.89189	2.2615	0.044319
**mmu-miR-19a-3p**	0	0	2207.505519	14374.22553	7.4763	0.0047744		2207.505519	14374.22553	0	0	-7.4565	0.0092767
**mmu-miR-451a**	0	0	3863.134658	5204.460967	6.6142	0.031333		3863.134658	5204.460967	0	0	-6.7432	0.049054

Exosomal MicroRNAs modulate the cognitive function in fasudil treated APPswe/PSEN1dE9 transgenic (APP/PS1) mice model of Alzheimer's disease

Yuqing Yan[1*#], Ye Gao[1*], Gajendra Kumar[3*#], Qingli Fang[1], Hailong Yan[1], Nianping Zhang[1], Yuna Zhang[1], Lijuan Song[2], Jiehui Li[1], Yucheng Zheng[1], Nan Zhang[1], Peijun Zhang[1], Cungen Ma[1, 2#]

1. Institute of Brain Science, Shanxi Key Laboratory of Inflammatory Neurodegenerative Diseases, Medical School of Shanxi Datong University, Datong, China.
2. The Key Research Laboratory of Benefiting Qi for Acting Blood Circulation Method to Treat Multiple Sclerosis of State Administration of Traditional Chinese Medicine, Research Center of Neurobiology, Shanxi University of Chinese Medicine, Taiyuan, China.
3. Department of Neuroscience, City University of Hong Kong, Kowloon, Hong Kong.

* Contributed equally.
# Corresponding author (Yuqing Yan: 779734216@qq.com; Gajendra Kumar: gajendra_kumar@brown.edu, , Cungen Ma: macungen@sxtcm.edu.cn).

Supplement table 4a: Prediction and analysis of target genes for WT_vs_ADNS.

S. no	miRNA	Gene list
1	mmu-let-7i-5p	Apc2, Armc2, Atp2b2, Col1a2, Cts8, Fbxl14, Gnl3l, Ifnar1, Il6, Lins1, Meis2, Msi2, Mup11, Mup17, Nf2, Nup214, Tnfrsf26, Zfp444
2	mmu-miR-126a-3p	Adam9, Dnmt1, Errfi1, Fzd7, Hoxa9, Irs1, Itga11, Pik3r2, Runx1, Sfpi1, Spred1, Ston1, Tal1, Vegfa
3	mmu-miR-140-3p	Fn1, Pou3f3, Smad2
4	mmu-miR-142a-3p	Adcy9, Akr1d1, Arntl, AW549877, Cd2ap, Egfl6, Hmgb1, Homez, Il6, Il6st, Irak1, Lnpk, Olr1, Sema6a, Shc4, Smarca2, Zfp949
5	mmu-miR-144-3p	4930539E08Rik, Abca1, Bsn, Cab39, Car10, Cdc42ep3, Cebpa, Enpp6, Gja5, Hdgfl3, Hif1a, Lifr, Lrig2, Map7d1, Mapre1, Prkaa1, Pten, Ptp4a3, Rcor3, Spred1, Sptbn1, Traf6, Uba1y, Unc5c, Zbtb34
6	mmu-miR-16-5p	Angel1, App, Arl2, Armcx6, Bcl11b, Bcl2, Bcl2l2, Bicd1, Bri3bp, Cadm1, Ccnd1, Ccne1, Ccnt2, Cd274, Cd40, Creb5, Ddx19b, Dhdh, Erlin2, Fbxo21, Fgd4, Fgf2, Idua, Irgq, Itgav, Jag1, Jun, Klc1, Lpcat2b, Mapkap1, Mdm4, Ncl, Pacsin2, Plpp3, Pomk, Rnf168, Slc6a4, Sorcs2, Spsb4, Tacc1, Tifab, Trim2, Vegfa, Wnt3a
7	mmu-miR-1903	A830018L16Rik, Agl, Baz1b, Btbd9, Cplx2, Dcc, Dgkg, Dusp26, Emp2, Gcm1, Gpnmb, Gpr146, Htr3b, Iigp1, Lpin1, Metap2, Nab1, Nbl1, Nhlh2, Ntm, Pbx2, Pigr, Prlr, Ptpro, Rab3c, Rgs17, Rs1, Sec14l3, Serpinb1c, Sh2d4a, Slc22a12, Slc7a13, Steap4, Synj2bp, Tango6, Tmprss11e, Tnfsf15, Trabd2b, Ttll7, Unc5d, Vash1, Zfp300
8	mmu-miR-19a-3p	Acadm, Akap2, Atp10a, Atxn1, Chrna7, Chrnd, Fam104a, Fam83d, Fgfr1op2, Fzd4, Gja3, Ilf3, Kdm2a, Lrp6, Map3k12, Nat8f2, Nat8f4, Nhsl1, Pten, PTEN, Rnf11, Slc35f1, Snap25, Snx27, Tet2, Timp2, Tmem25, Tnf, Ttc39b, Ubap2l, Zfpm2
9	mmu-miR-1a-3p	Acta1, Adar, Anxa5, Bdnf, Calm1, Calm2, Cdc42, Cdk9, Clcn3, Cpeb1, Ets1, Fn1, Fzd7, Gata4, Gja1, GTF2B, Hand2, Hdac4, Hes1, Hspa1b, Hspd1, Igf1, Igf1r, Irx5, Klf4, Map4k3, Mef2a, Meox2, Mkl1, Mup10, Mup11, Mup12, Mup14, Mup17, Mup19, Myh6, Myocd, Nfat5, Nppa, Pax3, Pax7, Pola1, Rarb, Rasa1, Rheb, Rnf170,

		Rps6, Sh3bgrl, Smarcb1, Smarcd2, Srf, Tgoln1, Tlx2, Ucp2
10	mmu-miR-1b-5p	1110059G10Rik, A630001G21Rik, Acot11, Acvr2a, Ado, AI429214, Akap7, Arl15, Atf2, Atp7a, B3gnt2, BC003965, Bpifc, Bsn, Casp8, Ccdc138, Ccdc169, Ccna2, Ccpg1, Cd28, Cds2, Chek1, Chic1, Cisd2, Csnk1g3, Csrnp3, Cwf19l2, Cysltr1, Dtna, Dzank1, E2f1, Ephb3, Etfbkmt, Fam169b, Fbxl14, Fbxo45, Foxk1, Frmd6, Gabpb2, Gabra2, Gfod1, Haus2, Hist1h1d, Hoxd1, Htt, Iars2, Il15ra, Iws1, Lhfpl4, Lin28b, Loxl3, Lrrc75b, Mavs, Mtf1, Nacc2, Nras, Nxpe3, Oip5, Pdxk, Pgm2l1, Pik3ca, Pitpnb, Piwil2, Plag1, Plxna1, Pofut1, Prl5a1, Prmt8, Prpf18, Psd3, Ptdss1, Rasa2, Rbbp9, Rbm41, Rbm47, Rgl1, Rps3, Rsbn1l, Rufy2, Sass6, Satb2, Sept3, Slc16a10, Slc16a9, Slc25a46, Slc41a3, Snx27, Srgap3, Ss18l1, St6galnac1, Strbp, Stxbp6, Tas1r3, Tbc1d13, Tbc1d22b, Tdrd1, Tfcp2l1, Tigd5, Tlr4, Tmem161b, Tmem200a, Tnrc6b, Tpte, Trhr, Ubac1, Unc5b, Uty, Wfdc12, Zbtb39, Zfp354a, Zfp446, Zfp462, Zfp874b, Zfp941, Zfp963, Zscan4c, Zscan4d
11	mmu-miR-25-3p	Btg2, Cd69, Chm, Cpeb1, Fam136a, Fbxw7, Gm5148, Il5ra, Ncam2, Rhbdl3, Stk10, Tagap, Tagap1, Uba1y, Wwp2, Xylt2, Zfp300, Zfp970, Zpbp
12	mmu-miR-451a	Abhd17b, Acc, Akt3, Ankrd17, Ankrd46, Arnt2, Atf2, Avl9, AW549877, Cab39, Clstn1, D14Abb1e, Ddx19b, Gdap1, Gm2a, Gnai3, Grin3a, Hey1, Ik, Ints10, Irf8, Klhl5, Limch1, Lonrf2, Lphn2, Lsm14a, Megf9, MIF, Myc, Nsmaf, Nucks1, Pom121, Ppm1l, Prkaa1, Rnf103, Scn8a, Setd5, Shank2, Sidt2, Snrk, Sptssa, Syp, Tenm2, Thbd, Thrsp, Trim33, Trpc3, Vezt, Xiap, Ywhaz, Zfp644

Exosomal MicroRNAs modulate the cognitive function in fasudil treated APPswe/PSEN1dE9 transgenic (APP/PS1) mice model of Alzheimer's disease

Yuqing Yan[1*#], Ye Gao[1*], Gajendra Kumar[3*#], Qingli Fang[1], Hailong Yan[1], Nianping Zhang[1], Yuna Zhang[1], Lijuan Song[2], Jiehui Li[1], Yucheng Zheng[1], Nan Zhang[1], Peijun Zhang[1], Cungen Ma[1, 2#]

1. Institute of Brain Science, Shanxi Key Laboratory of Inflammatory Neurodegenerative Diseases, Medical School of Shanxi Datong University, Datong, China.
2. The Key Research Laboratory of Benefiting Qi for Acting Blood Circulation Method to Treat Multiple Sclerosis of State Administration of Traditional Chinese Medicine, Research Center of Neurobiology, Shanxi University of Chinese Medicine, Taiyuan, China.
3. Department of Neuroscience, City University of Hong Kong, Kowloon, Hong Kong.

* Contributed equally.
# Corresponding author (Yuqing Yan: 779734216@qq.com; Gajendra Kumar: gajendra_kumar@brown.edu, , Cungen Ma: macungen@sxtcm.edu.cn).

Supplement table 4b: Prediction and analysis of target genes for ADNS_vs_ADF.

S. no	miRNA	Gene list
1	mmu-let-7i-5p	Apc2, Armc2, Atp2b2, Col1a2, Cts8, Fbxl14, Gnl3l, Ifnar1, Il6, Lins1, Meis2, Msi2, Mup11, Mup17, Nf2, Nup214, Tnfrsf26, Zfp444
2	mmu-miR-130b-3p	Acbd5, Arrdc3, Chrna7, Fgfr1op2, Homez, Inpp5e, Irf1, Kdm2a, Map3k12, Meox2, Mup13, Mup7, Nfia, Rhod, Snx27, Tgfb1, Timp2, Tmem25, Ubap2l
3	mmu-miR-19a-3p	Acadm, Atp10a, Atxn1, Chrna7, Chrnd, Fam104a, Fam83d, Fgfr1op2, Fzd4, Gja3, Ilf3, Kdm2a, Lrp6, Map3k12, Nat8f2, Nat8f4, Nhsl1, Pakap, PTEN, Pten, Rnf11, Slc35f1, Snap25, Snx27, Tet2, Timp2, Tmem25, Tnf, Ttc39b, Ubap2l, Zfpm2
4	mmu-miR-451a	Abhd17b, Acc, Akt3, Ankrd17, Ankrd46, Arnt2, Atf2, Avl9, AW549877, Cab39, Clstn1, D14Abb1e, Ddx19b, Gdap1, Gm2a, Gnai3, Grin3a, Hey1, Ik, Ints10, Irf8, Klhl5, Limch1, Lonrf2, Lphn2, Lsm14a, Megf9, MIF, Myc, Nsmaf, Nucks1, Pom121, Ppm1l, Prkaa1, Rnf103, Scn8a, Setd5, Shank2, Sidt2, Snrk, Sptssa, Syp, Tenm2, Thbd, Thrsp, Trim33, Trpc3, Vezt, Xiap, Ywhaz, Zfp644
5	mmu-miR-466i-5p	4921509C19Rik, Abcc9, Ackr4, Acot2, Acsm2, Acss1, Adamts14, Adamts17, Adarb2, Adgra1, Ado, Adra1b, Agtrap, Akap10, Akap2, Akap7, Ank2, Ap1ar, Apba1, Aplnr, Appl1, Aptx, Asah2, Asap3, Asb13, Ascl4, Asxl2, Atp2b3, Atxn1, B4galt6, Barhl2, Baz2b, Bcl2, Bcl2l11, Bend3, Bnc2, C5ar2, Cacna2d2, Calcoco1, Calcr, Camk1d, Cap2, Car10, Casc4, Cask, Casp8, Ccdc85a, Ccna2, Ccpg1, Cd1d1, Cd274, Cd28, Cd2ap, Cd33, Cd4, Cdh12, Cdh20, Cdk13, Cdk7, Cds2, Ceacam1, Ceacam18, Cer1, Chrnb4, Chrnd, Chst2, Clec7a, Clvs1, Cnih3, Cnksr2, Cntn3, Commd7, Cox15, Cradd, Creg2, Csf2ra, Cxcl15, D430042O09Rik, D630003M21Rik, D630045J12Rik, Ddx6, Dgkg, Dhdh, Dhfr, Dmd, Dmrta1, Dnaaf5, Dnase1l3, Dnlz, Draxin, Drp2, Dzank1, E2f8, Ebf3, Ece1, Efcab14, Egfl6, Ehd3, Ehhadh, Elfn1, Elovl6, Entpd1, Eogt, Epas1, Epha4, Epha7, Etv3, Evi2b, Fam107a, Fam169b, Fam198a, Fam213a, Fblim1, Fbrs, Fbxl17, Fech, Flnb, Flrt1, Foxk1, Frk, Frmd5, Fsd1l, Gabpb2, Gatc, Gcnt4, Gdpgp1, Gfod1, Gfpt1, Gfra2, Gm14137, Gm14326, Gm4841, Gm5615, Gna13, Gnal, Gnb4, Gramd1c, Grasp, Greb1, Gria3, Grid1, Grik3, Grk3, Gtf2h2, Has2, Havcr2, Hif1an, Hoxc8, Hpca,

		Hspb7, Htt, Iars, Iffo2, Ifi44, Igf1r, Igf2, Iglon5, Igsf11, Il18r1, Ildr2, Insig2, Insr, Iqgap2, Itga11, Itga9, Jarid2, Kank2, Kcnc1, Kcnip3, Kcnj1, Kcnj16, Kif1a, Klhl13, Klhl23, Kmt5b, Krt222, L1cam, Lbp, Lcp2, Lhx6, Lifr, Lin7a, Lnpk, Lpp, Lrrc32, Lrrc61, Lrrn4cl, Lrtm2, Ltf, Ly6g6c, Ly96, Maf, Mafb, Magee2, Man1c1, Man2a2, Map3k7, Mapk11, Mapkbp1, Mavs, Mbnl3, Mcidas, Mdm2, Mfap3l, Micall1, Mlec, Mocs1, Mon1b, Mrgpre, Ms4a5, Mtmr12, Muc13, Mup18, Mylk4, Nab1, Nab2, Napb, Nat8f2, Nat8f4, Nat8l, Nbeal1, Ncam1, Ncan, Nedd4l, Neu1, Neurod2, Nfat5, Nfatc1, Ngfr, Nid1, Nkap, Nkx2-9, Nmnat2, Npr3, Nqo2, Nr4a2, Nrbp2, Nsun3, Nwd1, Nxpe3, Oacyl, Onecut2, Opcml, Oprm1, Oxsm, Oxtr, P4ha3, Pacsin2, Palm2, Pappa2, Pcdh10, Pcdh17, Pclo, Pdcd4, Pdxk, Pgm5, Phactr3, Pik3r5, Pirt, Plcb1, Pld5, Podn, Pogk, Ppp1r16b, Ppp1r1c, Prdm12, Prdm8, Prelid3a, Prex2, Prkcd, Prkci, Proser3, Prrc2b, Prss42, Psd2, Psd3, Pstpip2, Ptgdr, Ptprb, Ptpre, Pura, Rab11fip1, Rab3c, Rab6b, Rab9b, Rabgap1, Rara, Rasa2, Rasal2, Rassf2, Reps2, Rfx3, Rgs9bp, Rhobtb1, Rhou, Rnasel, Rorb, Rpusd2, Rsph4a, Runx1, Runx1t1, Runx3, Saa4, Samd7, Scd3, Scn2a, Sema5a, Sema5b, Sema6a, Sfrp1, Sh2d2a, Shisa6, Shroom3, Sike1, Sim1, Six4, Ski, Slc17a5, Slc1a2, Slc22a8, Slc24a2, Slc25a12, Slc25a21, Slc30a10, Slc31a2, Slc39a14, Slc4a4, Slc5a8, Slc6a17, Slc6a6, Slc7a1, Slc8a1, Slc8a3, Smarca2, Smo, Smpd4, Snai2, Snap23, Snap25, Snx12, Sox1, Sp9, Srgap3, Ssr1, St18, St8sia1, Steap2, Stk10, Stxbp4, Stxbp5l, Supt7l, Syn3, Synj2bp, Syt15, Tacr1, Tacr2, Tbc1d30, Tbx15, Tbx22, Terf2ip, Tet2, Tfap2b, Tgfb2, Thsd4, Tiprl, Tmco1, Tmem132b, Tmem151b, Tmem236, Tmem245, Tmem26, Tmem47, Tmod2, Tnfrsf13c, Tns4, Trim65, Trp53i11, Trpc7, Trpm3, Tspan18, Tyw3, Ubtf, Uhrf1bp1l, Unc13b, Unc5d, Unc93a, Uncx, Urb2, Vdr, Vprbp, Vps33b, Vps37a, Vsnl1, Wars2, Wdr46, Wrn, Xk, Xpr1, Xrcc3, Zeb2, Zfand2a, Zfhx3, Zfp169, Zfp248, Zfp329, Zfp39, Zfp449, Zfp46, Zfp488, Zfp607b, Zfp641, Zfp68, Zfp691, Zfp74, Zfp92, Zfp931, Znrf3, Zscan29
6	mmu-miR-574-5p	A830018L16Rik, Asb7, Cdh12, Cpm, Cpne1, Ctsb, Dhdh, Fam213a, Fndc1, Gm5615, Gria3, Iffo2, Ifi44, Maoa, Mrap2, Mylk4, Piezo2, Ppp1cb, Rnasel, S1pr3, Sox1, Stxbp4, T2, Tbc1d5, Trim65, Tyw3, Ubac1, Zdhhc3, Zfp74, Zfp994
