# Supplementary figures and images for: Exosomal MicroRNAs modulate the cognitive function in fasudil treated APPswe/PSEN1dE9 transgenic (APP/PS1) mice model of Alzheimer’s disease
Source: Metab Brain Dis. 2024 Aug 1;39(7):1335–51. doi: 10.1007/s11011-024-01395-8 (PMC11513711; doi:10.1007/s11011-024-01395-8)

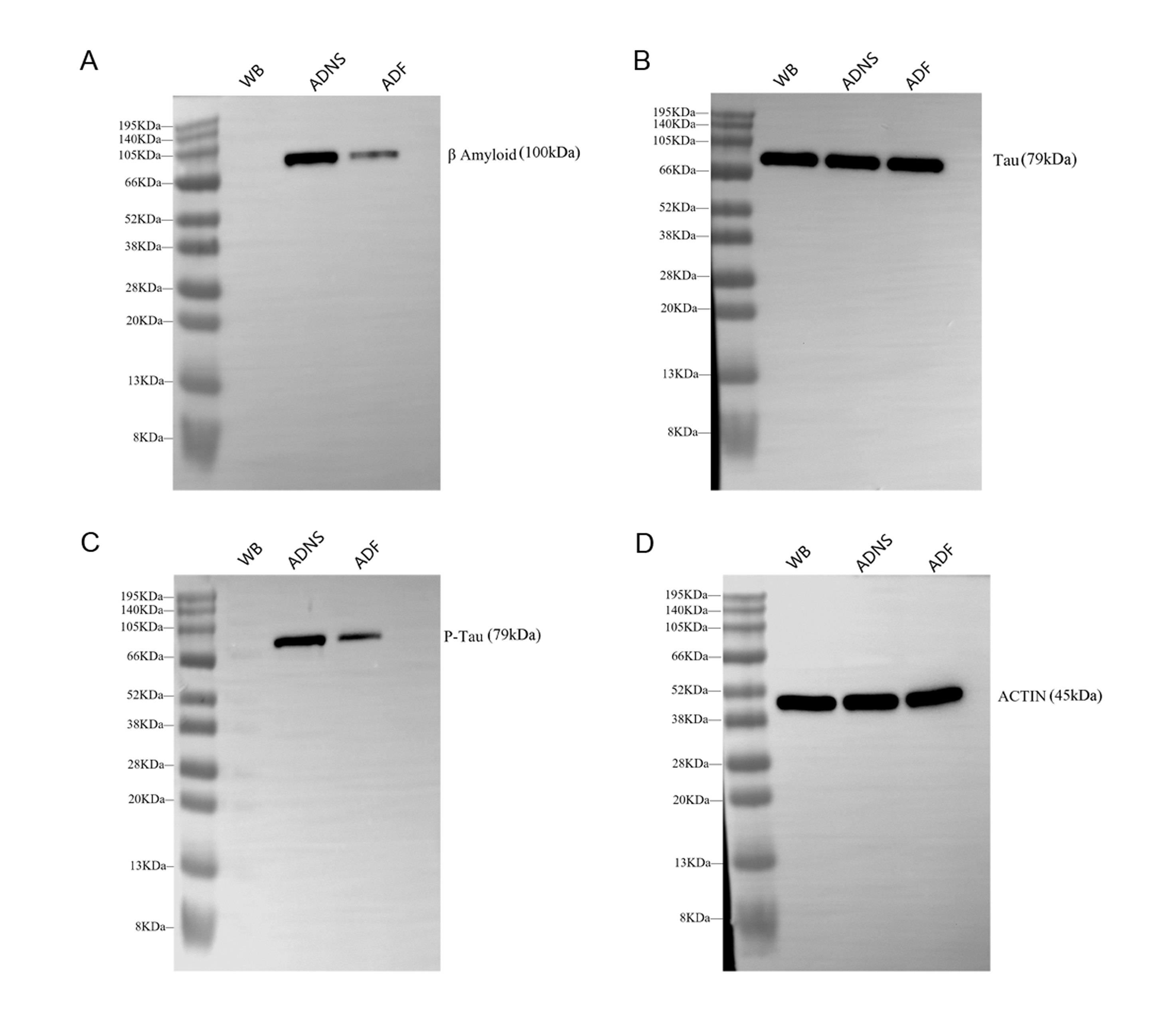

Supplement: Supplementary file 2 — Whole western blot picture of Aβ, Tau, phosphorylated Tau and beta actin. Whole western blot picture shows (A) β-amyloid (Aβ) (100 KDa), (B) Tau (79 KDa), (C) phosphorylated TAU (p-TAU) (79 KDa) and (D) beta actin (45 KDa). (PNG 718 kb) [file 11011_2024_1395_Fig8_ESM.png]

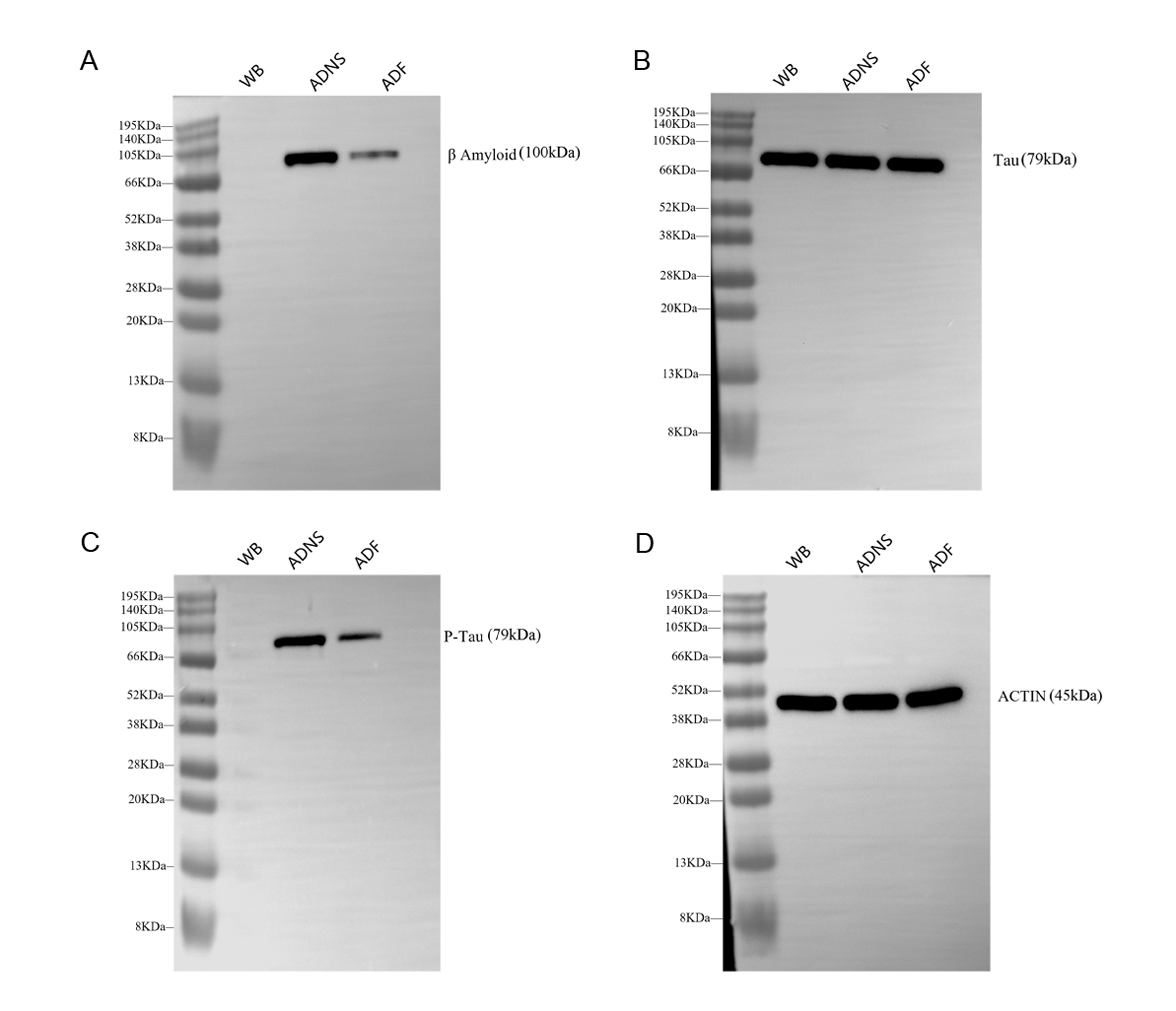

Supplement: Supplementary file 3 — High Resolution Image (TIF 8970 kb) [file 11011_2024_1395_MOESM2_ESM.tif]
